# Distinct brain atrophy progression subtypes underlie phenoconversion in isolated REM sleep behaviour disorder

**DOI:** 10.1016/j.ebiom.2025.105753

**Published:** 2025-05-29

**Authors:** Stephen Joza, Aline Delva, Christina Tremblay, Andrew Vo, Marie Filiatrault, Max Tweedale, Jean-François Gagnon, Ronald B. Postuma, Alain Dagher, Johannes Klein, Michele Hu, Petr Dusek, Stanislav Marecek, Zsoka Varga, John-Paul Taylor, John T. O'Brien, Michael Firbank, Alan Thomas, Paul C. Donaghy, Stéphane Lehéricy, Isabelle Arnulf, Isabelle Arnulf, Samir Bekadar, Eve Benchetrit, Alexis Brice, Vanessa Brochard, Alizé Chalançon, Benoit Colsch, Florence Cormier-Dequaire, Jean-Christophe Corvol, Virginie Czernecki, Bertrand Degos, Cécile Delorme, Pauline Dodet, Carole Dongmo-Kenfack, Marie-Odile Habert, Farid Ichou, Jonas Ihle, Cécile Galléa, Rahul Gaurav, Marie-Alexandrine Glachant, Manon Gomes, David Grabli, Elodie Hainque, Laetitia Jeancolas, Christelle Laganot, Stéphane Lehéricy, Suzanne Lesage, Smaranda Leu-Semenescu, Richard Levy, Valentine Maheo, Graziella Mangone, Louise Laure Mariani, Aurelie Méneret, Poornima Menon, Fanny Mochel, Vincent Perlbarg, Dijana Petrovska, Fanny Pineau, Nadya Pyatigorskaya, Sophie Rivaud-Pechoux, Emmanuel Roze, Sara Sambin, Julie Socha, Arthur Tenenhaus, Romain Valabregue, Marie Vidailhet, Lydia Yahia-Cherif, Marie Vidailhet, Jean-Christophe Corvol, ICEBERG Study Group, Richard Camicioli, Howard Chertkow, Simon Lewis, Elie Matar, Kaylena A. Ehgoetz Martens, Lachlan Churchill, Michael Sommerauer, Sinah Röttgen, Per Borghammer, Karoline Knudsen, Allan K. Hansen, Dario Arnaldi, Beatrice Orso, Pietro Mattioli, Luca Roccatagliata, Oury Monchi, Shady Rahayel

**Affiliations:** a The Neuro (Montreal Neurological Institute-Hospital), McGill University, Montreal, H3A 2B4, Canada; b Division of Neurology, Department of Medicine, and Neuroscience and Mental Health Institute, University of Alberta, Edmonton, Canada; c Centre for Advanced Research in Sleep Medicine, CIUSSS-NÎM – Hôpital du Sacré-Cœur de Montréal, Montreal, H4J 1C5, Canada; d Department of Psychology, Université du Québec à Montréal, Montreal, H2X 3P2, Canada; e Research Centre, Institut universitaire de gériatrie de Montréal, Montreal, H3W 1W5, Canada; f Department of Neurology, Montreal General Hospital, Montreal, H3G 1A4, Canada; g Oxford Parkinson's Disease Centre and Division of Neurology, Nuffield Department of Clinical Neurosciences, University of Oxford, Oxford, UK; h Department of Neurology and Centre of Clinical Neurosciences, First Faculty of Medicine, Charles University and General University Hospital, Prague, Czechia; i Translational and Clinical Research Institute, Newcastle University, Newcastle, UK; j Department of Psychiatry, University of Cambridge School of Clinical Medicine, Cambridge, UK; k Institut du Cerveau – Paris Brain Institute – ICM, Sorbonne Université, INSERM UMR 1127, CNRS 7225, Clinical Investigation Centre (CIC), Paris, 75013, France; l Department of Medicine (Neurology), University of Toronto, Toronto, Ontario, Canada; m Rotman Research Institute, Baycrest Health Services, Toronto, Ontario, Canada; n Parkinson's Disease Research Clinic, Macquarie Medical School, Macquarie University, Sydney, Australia; o Parkinson's Disease Research Clinic, Brain and Mind Centre, University of Sydney, Camperdown, NSW, 2050, Australia; p Department of Kinesiology and Health Sciences, University of Waterloo, Waterloo, N2L 3G1, Canada; q Centre of Neurology, Department of Parkinson, Sleep and Movement Disorders, University Hospital Bonn, Bonn, Germany; r German Centre for Neurodegenerative Diseases (DZNE), Bonn, Germany; s Department of Neurology, University Hospital Cologne, Faculty of Medicine, University of Cologne, Cologne, Germany; t Cognitive Neuroscience, Institute of Neuroscience and Medicine (INM-3), Forschungszentrum Jülich, Jülich, Germany; u Department of Nuclear Medicine and PET, Aarhus University Hospital, Aarhus, DK-8200, Denmark; v Department of Neuroscience, Rehabilitation, Ophthalmology, Genetics, Maternal and Child Health (DINOGMI), Clinical Neurology, University of Genoa, Genoa, 16132, Italy; w IRCCS Ospedale Policlinico San Martino, Genoa, 16132, Italy; x Department of Radiology, Radio-Oncology, and Nuclear Medicine, University of Montreal, Montreal, H3T 1A4, Canada; y Department of Medicine, University of Montreal, Montreal, H3T 1A4, Canada

**Keywords:** REM sleep behaviour disorder, Parkinson's disease, Dementia with Lewy bodies, MRI, Subtyping, Machine learning

## Abstract

**Background:**

Synucleinopathies include a spectrum of disorders varying in features and severity, including idiopathic/isolated REM sleep behaviour disorder (iRBD), Parkinson's disease (PD), and dementia with Lewy bodies (DLB). Distinct brain atrophy patterns may already be seen in iRBD; however, how brain atrophy begins and progresses remains unclear.

**Methods:**

A multicentric cohort of 1276 participants (451 polysomnography-confirmed iRBD, 142 PD with probable RBD, 87 DLB, and 596 controls) underwent T1-weighted MRI and longitudinal clinical assessments. Brain atrophy was quantified using vertex-based cortical surface reconstruction and volumetric segmentation. The unsupervised machine learning algorithm, Subtype and Stage Inference (SuStaIn), was used to reconstruct spatiotemporal patterns of brain atrophy progression.

**Findings:**

SuStaIn identified two distinct subtypes of brain atrophy progression: 1) a “cortical-first” subtype, with atrophy beginning in the frontal lobes and involving the subcortical structures at later stages; and 2) a “subcortical-first” subtype, with atrophy beginning in the limbic areas and involving cortical structures at later stages. Both cortical- and subcortical-first subtypes were associated with a higher rate of increase in MDS-UPDRS-III scores over time, but cognitive decline was subtype-specific, being associated with advancing stages in patients classified as cortical-first but not subcortical-first. Classified patients were more likely to phenoconvert over time compared to stage 0/non-classified patients. Among the 88 patients with iRBD who phenoconverted during follow-up, those classified within the cortical-first subtype had a significantly increased likelihood of developing DLB compared to PD, unlike those classified within the subcortical-first subtype.

**Interpretation:**

There are two distinct atrophy progression subtypes in iRBD, with the cortical-first subtype linked to an increased likelihood of developing DLB, while both subtypes were associated with worsening parkinsonian motor features. This underscores the potential utility of subtype identification and staging for monitoring disease progression and patient selection for trials.

**Funding:**

This study was supported by grants to S.R. from 10.13039/501100000143Alzheimer Society Canada (0000000082) and by Parkinson Canada (PPG-2023-0000000122).

The work performed in Montreal was supported by the Canadian Institutes of Health Research (CIHR), the Fonds de recherche du Québec - Santé (FRQS), and the W. Garfield Weston Foundation.

The work performed in Oxford was funded by 10.13039/501100000304Parkinson's UK (J-2101) and the 10.13039/501100013373National Institute for Health Research (NIHR) Oxford Biomedical Research Centre (BRC).

The work performed in Prague was funded by the 10.13039/501100009553Czech Health Research Council (grant NU21-04-00535) and by The National Institute for Neurological Research (project number LX22NPO5107), financed by the European Union – Next Generation EU. The work performed in Newcastle was funded by the NIHR Newcastle BRC based at Newcastle upon Tyne Hospitals NHS Foundation Trust and Newcastle University.

The work performed in Paris was funded by grants from the Programme d'investissements d'avenir (ANR-10-IAIHU-06), the Paris Institute of Neurosciences – IHU (IAIHU-06), the 10.13039/501100001665Agence Nationale de la Recherche (ANR-11-INBS-0006), Électricité de France (Fondation d’Entreprise EDF), the EU Joint Programme–Neurodegenerative Disease Research (JPND) for the Control-PD Project (Cognitive Propagation in Prodromal Parkinson's disease), the 10.13039/100022926Fondation Thérèse et René Planiol, the Fonds Saint-Michel; by unrestricted support for research on Parkinson's disease from Energipole (M. Mallart) and the Société Française de Médecine Esthétique (M. Legrand); and by a grant from the 10.13039/100007369Institut de France to Isabelle Arnulf (for the ALICE Study).

The work performed in Sydney was supported by a Dementia Team Grant from the 10.13039/501100000925National Health and Medical Research Council (#1095127).

The work performed in Cologne was funded by the 10.13039/501100003042Else Kröner-Fresenius-Stiftung (grant number 2019_EKES.02), the Köln Fortune Program, 10.13039/501100024583Faculty of Medicine, University of Cologne, and the “Netzwerke 2021 Program (10.13039/501100014690Ministry of Culture and Science of Northrhine Westphalia State).

The work performed in Aarhus was supported by funding from the 10.13039/501100003554Lundbeck Foundation, 10.13039/100008445Parkinsonforeningen (The Danish Parkinson Association), and the 10.13039/100010809Jascha Foundation.

**Research in context**

**Evidence before this study**

Idiopathic/isolated REM sleep behaviour disorder (iRBD) is a strong predictor for developing overt synucleinopathies including dementia with Lewy bodies (DLB) and Parkinson's disease (PD). Previous studies have established that patterns of brain atrophy in iRBD are already reminiscent of what is later seen in overt disease and are related to cognitive impairment, being associated with the development of DLB. However, how this brain atrophy begins and progresses remains unclear. To better understand the interindividual variability in iRBD and the distinct spatiotemporal patterns of neurodegenerative changes that lead to the development of overt disease, a systematic investigation of the sequential brain changes leading to overt disease is needed.

**Added value of this study**

This study includes the largest collection of structural brain MRI data of individuals with iRBD and leverages the powerful Subtype and Stage Inference (SuStaIn) machine learning algorithm to identify two distinct subtypes of brain atrophy progression: a “cortical-first” subtype and a “subcortical-first” subtype. We demonstrate that these brain atrophy progression subtypes are associated with different disease trajectories, with patients classified into the cortical-first subtype having an increased likelihood of developing DLB compared to PD, while both subtypes are associated with worsening parkinsonian motor features over time. This study, for the first time, delineates the subtypes of brain atrophy progression in iRBD and their distinct associations with clinical outcomes.

**Implications of all the available evidence**

The identification of distinct atrophy progression subtypes has significant implications for the early identification of disease trajectories in iRBD. This stratification can help guide patient selection for clinical trials, potentially improving outcomes by targeting therapies based on the underlying atrophy subtype. As synucleinopathies advance, understanding the spatial and temporal dynamics of brain atrophy will be critical for prognostication and early intervention in patients with iRBD.

## Introduction

Synucleinopathies are pathologically defined by the misfolding and aggregation of alpha-synuclein.^1^ During the prodromal phases of disease, patients manifest a variety of deficits in multiple clinical domains, including cognitive and motor abnormalities, olfactory dysfunction, constipation, dysautonomia, and sleep disorders.^2^ One highly studied prodromal phenotype is idiopathic/isolated REM sleep behaviour disorder (iRBD), a parasomnia characterised by dream enactment behaviours during REM sleep.^3^ The vast majority of patients with iRBD will eventually develop an overt and clinically-defined disorder, mainly dementia with Lewy bodies (DLB) and Parkinson's disease (PD), and less frequently multiple system atrophy.^4^

As a prodromal synucleinopathy, clinical changes and patterns of brain atrophy in iRBD are already reminiscent of what is seen in overt disease.^4^, ^5^, ^6^ In particular, patients with iRBD and concomitant mild cognitive impairment have more extensive cortical and subcortical abnormalities compared to those without mild cognitive impairment, with the severity of atrophy predicting subsequent development of DLB.^7^, ^8^, ^9^ This supports the notion that substantial variability exists between patients with iRBD during this prodromal phase, with some destined to develop dementia earlier in their disease course than others.^10^ *In silico* modelling of atrophy in iRBD, based on computational spreading models of alpha-synuclein,^11^, ^12^, ^13^ has demonstrated that gene expression and structural connectivity jointly influence brain neurodegeneration.^5^ Notably, a closer match between the *in silico* atrophy pattern and the patient's actual atrophy pattern correlates with increased cognitive impairment but not motor impairment in iRBD.^5^ Identifying patterns in this variability might be useful for prognostic purposes and allow more precise selection of patients for future therapeutic trials.^3^ However, the changes in brain morphology that begin during iRBD and eventually progress toward the development of dementia remain unclear.

To better understand the relationships between interindividual variability within patients with iRBD and their subsequent transition to dementia and parkinsonism, a systematic investigation of the specific sequential brain changes leading to DLB and PD is needed. Several studies have documented the longitudinal brain changes taking place over time in iRBD,^9^^,^^14^^,^^15^ but these have been restricted by a limited number of patients, the high level of inter-assessment variability in imaging techniques, and the extended follow-up delay between the diagnosis of iRBD and phenoconversion.

In this study, we performed a comprehensive quantification of brain atrophy in iRBD, PD, and DLB and reconstructed the subtypes of spatiotemporal changes in brain atrophy progression from cross-sectional data to understand their associations with clinical disease progression. We compiled the largest collection of structural brain MRI data acquired to date in patients with iRBD (n = 451 from 11 international study centres). To derive atrophy-driven subtypes of iRBD and their associated patterns of progression, we performed vertex-based cortical surface analysis of thickness and subcortical volume quantification on the complete dataset and applied the Subtype and Stage Inference (SuStaIn) model, an unsupervised machine learning algorithm that uses a combined disease progression modelling and clustering approach on cross-sectional scans of patients at different stages of a clinical continuum.^16^ Finally, we describe the clinical characteristics and phenoconversion status of the resulting data-driven subtypes to gain an understanding of the relationship between patterns of atrophy in iRBD and the development of dementia and parkinsonism.

## Methods

### Participants

**Fig. 1:**
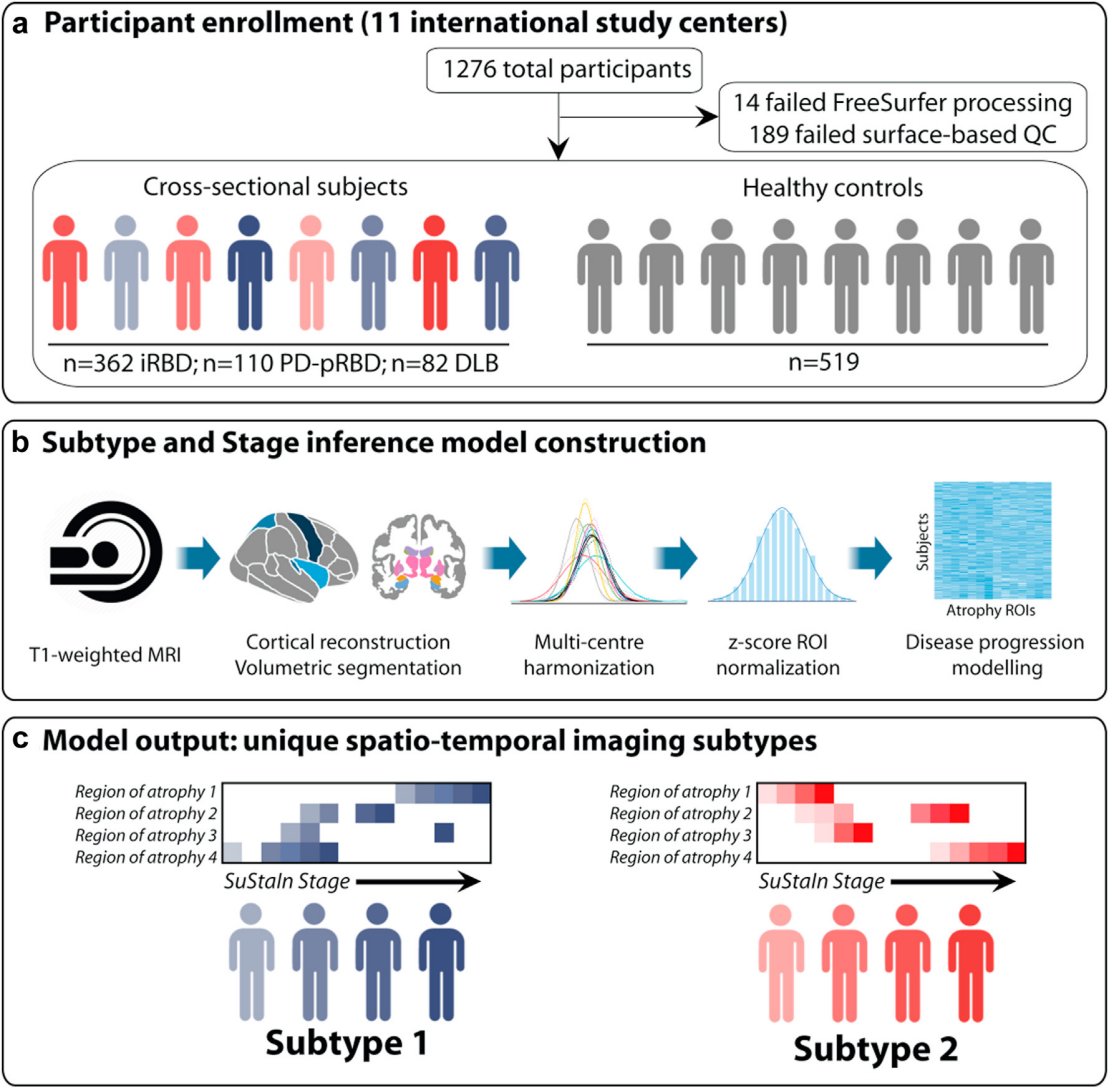
**Overview of study design, data processing, and subtype modelling**. **(a)** 1276 participants were recruited, with 203 participants excluded after quality control. **(b)** T1-weighted MRI scans underwent cortical reconstruction, volumetric segmentation, multi-centre harmonisation, z-score normalisation, and disease progression modelling using the SuStaIn algorithm. **(c)** SuStaIn identified two distinct subtypes of brain atrophy progression, each with unique spatiotemporal patterns and stages of disease. DLB = dementia with Lewy bodies; iRBD = idiopathic/isolated REM sleep behaviour disorder; PD-pRBD = Parkinson's disease with probable RBD; QC = quality control; ROI = region of interest.

A total of 1276 participants were prospectively recruited for this study and underwent T1-weighted brain MRI imaging and clinical assessments (see Fig. 1 for an overview of the study protocol). Of these, 451 had polysomnography-confirmed iRBD, 142 had PD with probable RBD (PD-pRBD), 87 had DLB, and 596 were healthy controls recruited in every centre. Biological sex was collected by self-report of the study participants.

Participant recruitment by study centre and disease group are detailed in Supplementary Table S1. Participants were recruited from the Centre for Advanced Research in Sleep Medicine at the Hopital du Sacre-Coeur de Montreal and The Neuro (n = 178), the Oxford Parkinson's Disease Centre (n = 147), the Department of Neurology at Charles University (n = 140), Newcastle University (n = 135), the Movement Disorders clinic at the Hôpital de la Pitié-Salpêtrière (n = 130), the COMPASS-ND Study from the Canadian Consortium on Neurodegeneration in Ageing (CCNA; n = 71),^17^, ^18^, ^19^ the Parkinson's Disease Research Clinic at the University of Sydney (n = 56), the Department of Neurology at the University of Cologne (n = 47), Aarhus University Hospital (n = 38), the IRCCS Ospedale Policlinico San Martino in Genoa (n = 29), as well as part of the Parkinson's Progression Markers Initiative (PPMI; n = 305).^20^ A subset of the patients with iRBD (n = 182, 40%) included in this study were part of previous studies investigating prodromal atrophy in synucleinopathies.^5^^,^^6^ Patients with iRBD were diagnosed using the International Classification of Sleep Disorders, third edition diagnostic criteria,^21^ including video-polysomnography, and underwent clinical assessments to confirm absence of DLB, Parkinson's disease, and multiple system atrophy at the closest examination in time to the MRI acquisition. Patients with iRBD were followed longitudinally approximately every 6–12 months in every centre to assess for the development of dementia and parkinsonism (phenoconversion). Clinical assessments used at all sites included cognitive testing with either the Montreal Cognitive Assessment (MoCA) or the Mini-Mental State Examination (MMSE), and motor examination using the Movement Disorders Society—Unified Parkinson's Disease Rating Scale, part III (MDS-UPDRS-III) or the original 1987 version (UPDRS-III). Patients with probable DLB were diagnosed using previously published criteria.^22^ Patients with PD-pRBD were recruited from the PPMI baseline cohort and the presence of probable RBD was defined by a cut-off score ≥5 on the RBD Screening Questionnaire.^23^

### MRI acquisition and processing

Structural T1-weighted brain MRI scans were acquired at each site and are detailed in Supplementary Table S1. T1-weighted scans underwent cortical reconstruction and volumetric segmentation using the FreeSurfer 7.1.1 image analysis suite (http://surfer.nmr.mgh.harvard.edu). The technical details of the FreeSurfer procedure have been described previously.^5^ Briefly, this processing included motion correction, removal of non-brain tissue using a hybrid watershed/surface deformation procedure, automated Talairach transformation, segmentation of the subcortical white matter and deep grey matter volumetric structures, intensity normalisation, tessellation of the grey matter white matter boundary, automated topology correction, and surface deformation following intensity gradients to optimally place the grey/white and grey/CSF borders at the location where the greatest shift in intensity defines the transition to the other tissue class. Once the cortical models were complete, deformable procedures were performed including surface inflation, registration to a spherical atlas based on individual cortical folding patterns to match cortical geometry across patients, parcellation of the cerebral cortex into units with respect to gyral and sulcal structure, and creation of a variety of surface-based data. This method used both intensity and continuity information from the entire MRI volume in segmentation and deformation procedures to produce representations of cortical thickness, calculated as the closest distance from the grey/white boundary to the grey/CSF boundary at each vertex on the tessellated surface. The maps were created using spatial intensity gradients across tissue classes and were therefore not simply reliant on absolute signal intensity.

All surface maps were inspected visually by a trained rater (S.R.) and scored from 1 to 4 based on published guidelines.^24^^,^^25^ Scans with major artefacts or reconstruction errors (score >2) were excluded from further analyses. Due to the significant atrophy found on DLB scans and the impact on surface reconstruction, the cortical surfaces from patients with DLB and associated controls were manually edited slice-by-slice (S.J., S.R., A.De.) and reprocessed. Cortical thickness, cortical volume, and subcortical volume measurements were next extracted from the resulting maps using the bilateral 83-region Desikan–Killiany atlas (68 cortical regions and 15 subcortical regions, namely the bilateral thalamus, caudate, putamen, pallidum, hippocampus, amygdala, nucleus accumbens, and brainstem). These metrics were all extracted because they were shown to be differentially affected in iRBD.^5^^,^^26^ Given that volume scales with head size,^27^ volume values were normalised by dividing values by the estimated total intracranial volume. To reduce the number of input features when modelling subtypes and preserve sufficient power, the labels of each individual parcellation were fused together inside FreeSurfer to yield lobar measurements of cortical thickness for the frontal, parietal, temporal, occipital, and cingulate lobes, as done previously.^16^^,^^28^^,^^29^ To control for the differences in scanner acquisitions, we next applied NeuroComBAT on the regional measurements, a batch-correcting tool widely used in multisite MRI studies that removes scanner-dependent variations while preserving the biological variance of interest, using age, sex, and disease group as covariates.^30^, ^31^, ^32^ Although the CCNA and PPMI cohorts involved multiple different scanners, these cohorts were each included as single entities in the NeuroComBAT harmonisation process, since i) the CCNA participants were scanned using the harmonised Canadian Dementia Imaging Protocol, which was developed to ensure consistency in MRI acquisitions across multiple centres^33^; and ii) the site details of PPMI participants are not available due to confidentiality restrictions. For harmonisation, we expressed each regional measurement as a piecewise linear *z*-score normalised to the control population using age and sex as regression covariates as previously described (see Supplementary Table S2 for group descriptives).^16^ This allowed the brain measurements from each patient to reflect deviations from what was expected for age and sex, thereby ensuring that the identified progression patterns were not merely reflective of normal ageing. Regions of interest were averaged between hemispheres; paired t-tests between left and right regions determined that there was no statistically significant difference between them (all p-values > 0.084). The NeuroComBAT-corrected, *z*-scored regional measurements served as the input for the analyses involving the reconstruction of transdiagnostic brain atrophy subtypes in synucleinopathies.

### Brain atrophy subtype and stage inference modelling

To reconstruct brain atrophy subtypes and stages from cross-sectional imaging data, we used the SuStaIn algorithm implemented in Python.^16^^,^^34^ In contrast to conventional analyses, which would generate subtypes exclusively based on temporal progression, the SuStaIn algorithm considers both temporal and spatial information in order to define synucleinopathy groups with distinct patterns of progression (subtypes) and assigns a disease stage for each participant, thereby allowing for the identification of transdiagnostic trajectories of brain neurodegeneration. We ran SuStaIn using 25 start points and 1,000,000 Markov Chain Monte Carlo iterations. The optimal number of subtypes was determined using the cross-validation information criterion calculated through 10-fold cross-validation.^16^ The SuStaIn algorithm subtyped individuals by calculating the maximum likelihood they belong to each subtype, and staged individuals by calculating their average stage weighted by the probability they belonged to each stage of each subtype. Individuals that were assigned a stage of 0 were determined to be “non-classifiable”, whereas individuals with a higher probability of belonging to a SuStaIn subtype were determined to be “classifiable”. To compare the subtype progression patterns between different neuroimaging metrics (i.e., cortical thickness *vs*. cortical volume) and across cross-validation folds (i.e., the cross-validation similarity metric), we calculated the Bhattacharyya coefficient^35^ between the position of each biomarker event in the two subtype progression patterns, averaged across biomarker events and Markov Chain Monte Carlo samples, as previously described.^16^ To ensure the robustness of our subtypes, we repeated the same analyses in the groups of patients with iRBD or DLB alone and in the group of patients with iRBD alone. The Bhattacharrya coefficient^35^ was used to assess the similarity of these brain atrophy progression patterns compared to the initial model involving patients with iRBD, DLB, and PD-pRBD. SuStaIn models were visualised using Brainpainter software.^36^

### Statistical analyses

Statistical analyses were performed in R (version 4.3.2). MMSE scores were converted to MoCA scores, which involved 73 patients with DLB and 18 patients with iRBD.^37^ UPDRS-III scores were converted to MDS-UPDRS-III scores in 43 patients with iRBD as previously described.^4^ Demographics and clinical variables were compared between patients and controls using ANOVA with post-hoc Tukey HSD testing and χ^2^ testing with post-hoc pairwise comparisons. Comparisons between subtypes used t-tests for continuous variables and χ^2^ tests for categorical variables. The progression of clinical variables with respect to SuStaIn subtypes and stages was assessed by linear regression using age, sex, SuStaIn subtype, SuStaIn stage, the interaction between subtype and stage, and the probability of subtype as covariates (i.e., clinical variable ∼ age + sex + subtype + stage + interaction between subtype and stage + probability of subtype). Logistic regression was also used to predict the log-odds of phenoconversion (i.e., received a clinical diagnosis at the last follow-up compared to being phenoconversion-free) and log-odds of phenoconversion outcomes in iRBD (DLB compared to PD) with respect to SuStaIn subtypes (i.e., phenoconversion ∼ age + sex + SuStaIn subtype + SuStaIn stage + interaction between subtype and stage). We did not include “years after iRBD onset” in the logistic regression models because the precise onset of iRBD symptoms is often uncertain and subject to recall bias. When interactions were significant, partial correlations were used for assessing subtype-specific associations while controlling for other covariates part of the logistic model.

### Ethics approval and consent to participate

Ethics approval was obtained from the local institutional boards of each centre with subject consent in accordance with the Declaration of Helsinki. The current study was approved by the Research Ethics Board of the McGill University Health Centre (MP-37-2022-7744) and the Quebec Integrated University Centre for Health and Social Services of Northern Island of Montreal (MEO-37-2024-2699).

### Role of funders

The sources of funding did not influence the design of the study, the collection of data, the analysis of data, the interpretation of results, or the writing of the manuscript.

## Results

### Participant demographics

Of the 1276 participants with T1-weighted imaging, 14 (1.1%) failed the FreeSurfer processing step and 189 (14.8%) did not pass surface-based quality control, leading to a final sample for analysis of 362 patients with iRBD, 110 with PD-pRBD, 82 with DLB, and 519 controls. As expected, patients with DLB were older (76.8 ± 6.45 years), had lower MoCA scores (14.4 ± 5.46), and higher MDS-UPDRS-III scores (32.1 ± 18.1) compared to the iRBD, PD-pRBD, and control groups. Patients with iRBD were younger (67.1 ± 6.95 years), with intermediate MoCA (25.7 ± 3.02) and MDS-UPDRS-III scores (6.04 ± 5.57). Controls were slightly younger than patients with iRBD (65.6 ± 10.1 years) and had the highest MoCA scores (26.8 ± 2.36) and lowest MDS-UPDRS-III scores (2.28 ± 4.44). Finally, patients with PD-pRBD were the youngest (62.1 ± 8.93), had comparable MoCA scores as patients with iRBD (25.9 ± 3.31), and higher MDS-UPDRS-III scores compared with patients with DLB (21.4 ± 9.61). Summarised demographic and clinical information is available in Supplementary Table S2 and Supplementary Figure S1.

### SuStaIn identifies two brain atrophy subtypes

**Fig. 2:**
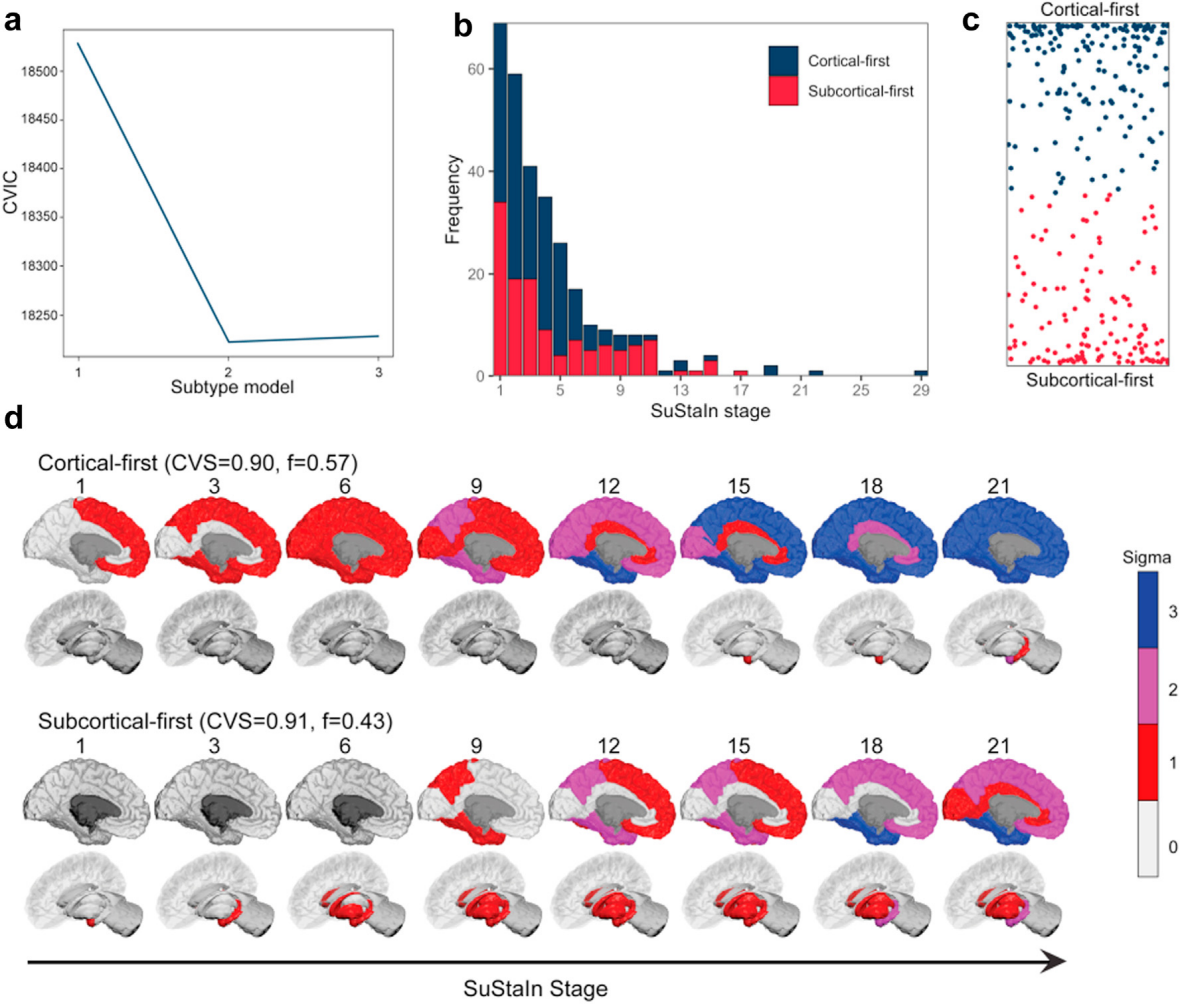
**SuStaIn identified a two-subtype model as being the best representation of brain atrophy progression in patients**. **(a)** CVIC across 10-fold cross-validation of left-out individuals; lower CVIC represents better model fit. **(b)** Distribution of subtypes across SuStaIn stages. **(c)** The assignability of disease subtype, operationalised as the distance from the top or bottom axis, which represents the maximum probability (100%) of that subtype. **(d)** SuStaIn identified two unique subtypes of brain atrophy progression. At each stage, the colour in each region indicates the level of severity of atrophy, with grey representing unaffected regions, red mildly affected regions (z-score of −1), magenta moderately affected regions (z-score of −2), and blue severely affected regions (z-score of −3 or more). Brainstem atrophy begins at approximately stage 6 in the subcortical-first subtype (not shown). CVIC = cross-validation information criterion; CVS = cross-validation similarity; iRBD = idiopathic/isolated REM sleep behaviour disorder; SuStaIn = Subtype and Staging Inference.

First, we used SuStaIn to identify subtypes of brain atrophy progression in the neurodegenerative spectrum linking iRBD, PD-pRBD, and DLB. Using cortical thickness and subcortical volume regions of interest as input (Supplementary Table S3), SuStaIn identified a two-subtype model as being the best representation of brain atrophy progression in patients (Fig. 2a). This subtyping classified 304 (55%) patients with iRBD, PD-pRBD, or DLB into one of the two subtypes (Fig. 2b and c), each with distinct sequences of atrophy (Fig. 2d): (i) a “cortical-first” progression subtype, found in 58% of classifiable patients, characterised by atrophy beginning in the frontal lobes followed by the temporal and parietal areas and remaining cortical areas, with the involvement of subcortical structures at later stages; and (ii) a “subcortical-first” progression subtype, found in 42% of classifiable patients, characterised by atrophy beginning in the limbic areas (primarily the amygdala and hippocampus), followed by structures of the basal ganglia and only involving cortical structures at later stages. The cortical-first subtype included 177 patients, namely 111 (62.7%) iRBD, 33 PD-pRBD (18.6%), and 33 (18.6%) DLB, while the subcortical-first subtype included 127 patients, namely 75 (59.1%) iRBD, 22 PD-pRBD (17.3%), and 30 (23.6%) DLB (Table 1). The remaining 250 patients with synucleinopathies (176 [70.4%] iRBD, 55 PD-pRBD [22%], and 19 [7.6%] DLB) were categorised as stage 0/non-classifiable (i.e., assigned to very early SuStaIn stages at which point there is low confidence in the subtype assignment or displayed a different atrophy pattern compared to the rest of the sample).

**Table 1:** Baseline demographic and clinical variables for each brain atrophy progression subtype.

Phenoconversion	Classifiable			Subtyped		
	Non-classifiable	Classifiable	p-value^a^	Cortical-first	Subcortical-first	p-value^b^
**Demographics**						
n (%): iRBD	176 (70.4)	186 (61.2)	0.710	111 (62.7)	75 (59.1)	0.061
n (%): DLB	19 (7.6)	63 (20.7)	**<0.001**	33 (18.6)	30 (23.6)	0.789
n (%): PD-pRBD	55 (22)	55 (18.1)	1.0	33 (18.6)	22 (17.3)	0.292
Age: All	67.1 (8.2)	68 (8.7)	0.218	68.1 (8.8)	67.7 (8.7)	0.654
Age: iRBD	67.3 (7.3)	67 (6.6)	0.667	67.3 (6.4)	66.5 (7.1)	0.472
Age: DLB	75.9 (6)	77.1 (6.6)	0.48	78.3 (6.9)	75.7 (6)	0.116
Age: PD-pRBD	63.3 (9)	60.9 (8.8)	0.151	61 (8.9)	60.7 (8.8)	0.921
% male/% female	83.8/16.2	83.8/16.2	0.960	82.7/17.3	85.6/14.4	0.611
Stage^c^ (SD): All	0 (0)	4.3 (3.9)	**<0.001**	4.0 (3.8)	4.6 (3.9)	0.232
Stage^c^ (SD): iRBD	0 (0)	3.6 (2.8)	**<0.001**	3.2 (1.9)	4.2 (3.6)	**0.029**
Stage^c^ (SD): DLB	0 (0)	6.7 (6)	**<0.001**	7.5 (7)	5.7 (4.6)	0.215
Stage^c^ (SD): PD-pRBD	0 (0)	3.8 (2.8)	**<0.001**	3.4 (2)	4.3 (3.7)	0.293
**Clinical variables**						
MDS-UPDRS-III (SD): All	10.4 (11.1)	15.4 (15.2)	**<0.001**	15.8 (15.9)	14.8 (14.3)	0.56
MDS-UPDRS-III (SD): iRBD	5.2 (4.9)	6.9 (6.1)	**0.005**	7.1 (6.6)	6.5 (5.4)	0.475
MDS-UPDRS-III (SD): DLB	24.4 (17.4)	34.4 (17.8)	**0.036**	37.4 (18.9)	31.2 (16.2)	0.166
MDS-UPDRS-III (SD): PD-pRBD	21.7 (9.8)	21.1 (9.4)	0.737	21.6 (9.2)	20.4 (9.9)	0.634
MoCA (SD): All	25.4 (3.7)	23.3 (6)	**<0.001**	23.3 (6.3)	23.3 (5.6)	0.984
MoCA (SD): iRBD	26.1 (2.7)	25.4 (3.2)	**0.044**	25.4 (3.4)	25.4 (3)	0.921
MoCA (SD): DLB	17.5 (5.2)	13.5 (5.3)	**0.015**	12.6 (5.9)	14.6 (4.2)	0.152
MoCA (SD): PD-pRBD	25.7 (3.1)	26 (3.5)	0.675	25.9 (3.4)	26.1 (3.8)	0.836
% MCI: iRBD^d^	36.3	45.3	0.084	43.0	48.6	0.452
% MCI: PD-pRBD^d^	40.0	36.7	0.745	37.9	35.0	0.834

Bold values represent significant p-values.

DLB = dementia with Lewy bodies; iRBD = idiopathic/isolated REM sleep behaviour disorder; MoCA = Montreal Cognitive Assessment; MCI = mild cognitive impairment; MDS-UPDRS-III = Movement Disorders Society – Unified Parkinson's Disease Rating Scale, Part III; MoCA = Montreal Cognitive Assessment; PD-pRBD = Parkinson's disease with probable REM sleep behaviour disorder; SD = standard deviation; SuStaIn = Subtype and Staging Inference.

a Non-classifiable group *vs*. classifiable group; t-tests for continuous variables and χ2 tests for categorical variables.

b Cortical-first subtype *vs*. subcortical-first subtype; t-tests for continuous variables and χ2 tests for categorical variables.

c Stage refers to SuStaIn stage.

d MCI as defined by ≤ 25/30 on MoCA; all patients with DLB met criteria for dementia.

The average similarity between cross-validation folds was >90% for each subtype, indicating high reliability of subtype progression patterns with 10-fold cross-validation. Moreover, the identification of two distinct subtypes was recapitulated when using cortical volume (as a measure of cortical atrophy instead of cortical thickness) with subcortical volume as input features, with >86% similarity when comparing the subtypes' progression patterns (Supplementary Figure S2). Since atrophy has previously been reported to be more prominent in iRBD associated with MCI, and given that atrophy predicts the development of DLB compared to PD in iRBD,^5^^,^^7^^,^^8^ we hypothesised that the more extensive atrophy observed in patients with DLB may influence the subtyping results. Therefore, to test the robustness of our subtyping, we performed secondary analyses excluding patients with PD-pRBD from the SuStaIn modelling and, separately, using only patients with iRBD as inputs (Supplementary Figure S3 and Supplementary Table S4). In both cases, the two subtypes identified in the primary SuStaIn model were recapitulated with similar patterns of progression. As expected, the iRBD-only model showed increased uncertainty at higher stages, particularly in the cortical-first subtype. The exclusion of patients with PD-pRBD resulted in a distribution of classifiable patients comparable to the main SuStaIn model. The Bhattacharrya coefficient indicated a similarity between 81% and 94% with the original model that included patients with iRBD, PD-pRBD, and DLB. Taken together, this indicates that the primary driver of subtyping and staging reflects the progression of cortical and subcortical atrophy, independent of the inclusion of patients with PD-pRBD or the use of patients with iRBD alone.

**Fig. 3:**
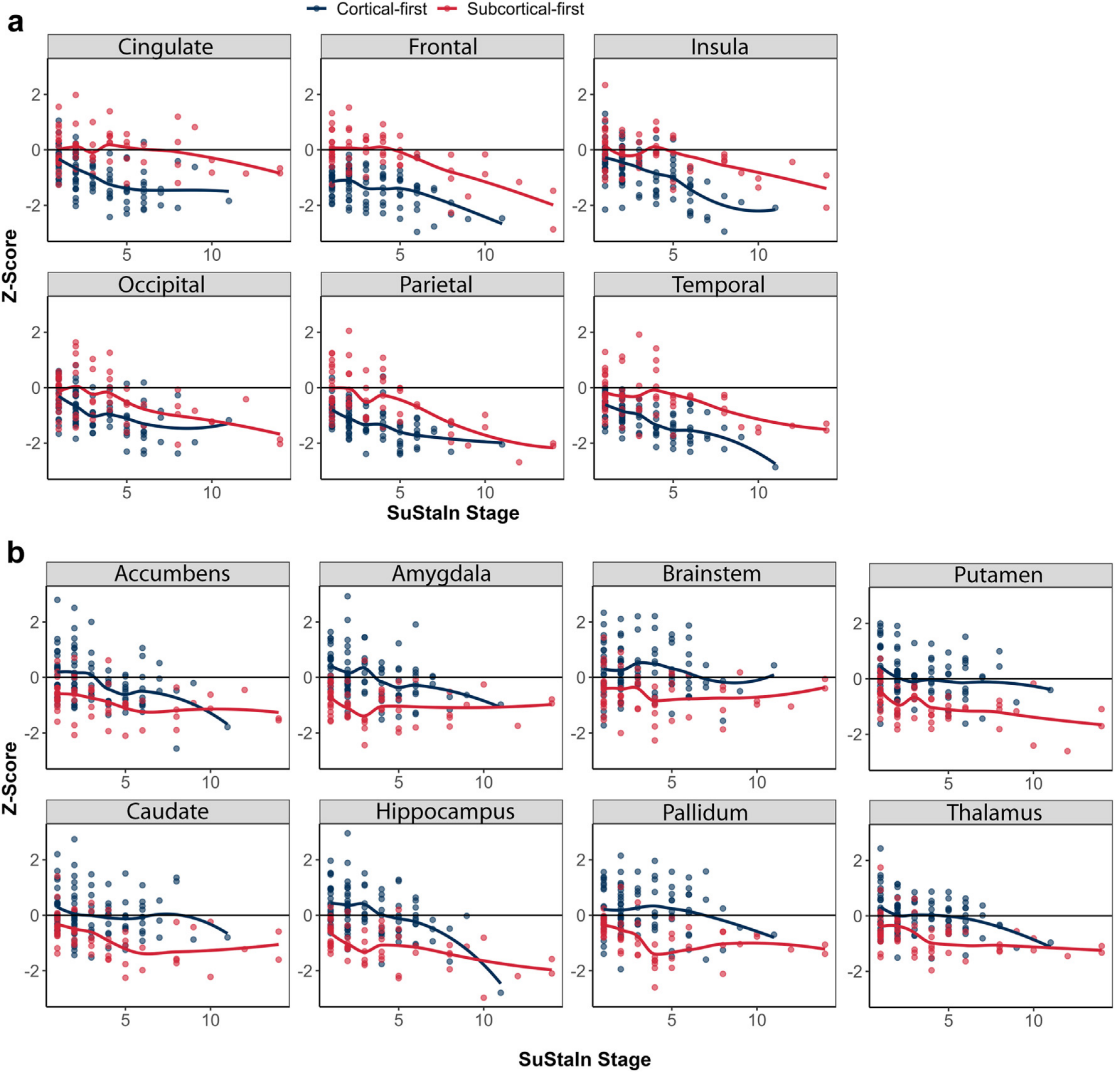
**Progression of cortical and subcortical atrophy by subtype and stage in iRBD**. The progression of atrophy in cortical regions **(a)** and subcortical regions **(b)** used in the SuStaIn modelling in classifiable patients with iRBD. iRBD = idiopathic/isolated REM sleep behaviour disorder; SuStaIn = Subtype and Staging Inference.

Inspecting the subtypes based on the progression of atrophy in each brain region revealed that compared to normative data from control scans, patients with iRBD from the subcortical-first subtype had rapid subcortical volume loss in the early stages, with relative stability of most cortical structures but progressive atrophy of the hippocampus, putamen, and cortical structures at later stages (Fig. 3). This pattern was generally reversed in cortical-first patients, where atrophy of cortical structures occurred in the earlier stages followed by relative stability in the cingulate, occipital, and parietal structures, with progressive atrophy in the frontal, insular, and temporal cortical areas and subcortical structures (Fig. 3).

### Atrophy subtypes are related to increased clinical burden

Next, we investigated whether demographics and clinical variables differed between classifiable and stage 0/non-classifiable patients and between the identified subtypes. The baseline demographics and clinical variables of the classifiable and stage 0/non-classifiable groups are shown in Table 1. The classifiable group (which includes patients identified as either cortical-first or subcortical-first subtypes) had more patients with DLB (20.7% *vs*. 7.6%, p < 0.001 [χ^2^ test]) and had worse MoCA (23.3 ± 6.0 *vs*. 25.4 ± 3.7, p < 0.001 [t-test]) and MDS-UPDRS-III (15.4 ± 15.2 *vs*. 10.4 ± 11.1, p < 0.001 [t-test]) scores than stage 0/non-classifiable patients. Worse clinical scores in classifiable patients were also observed when comparing within iRBD and DLB groups. In other words, our modelling identified brain atrophy subtypes related to higher cognitive and motor disease burden.

In contrast, there were no significant differences in sex proportion, age, MoCA scores, and MDS-UPDRS-III scores when comparing patients classified in the cortical-first versus the subcortical-first atrophy progression subtypes (Table 1). However, it is important to note that these comparisons are based on group means, and the groups represent patients at different SuStaIn stages on the atrophy progression subtype, limiting the interpretability of these findings due to confounding effects of disease progression.

### Brain atrophy severity relates to cognitive and motor progression

**Fig. 4:**
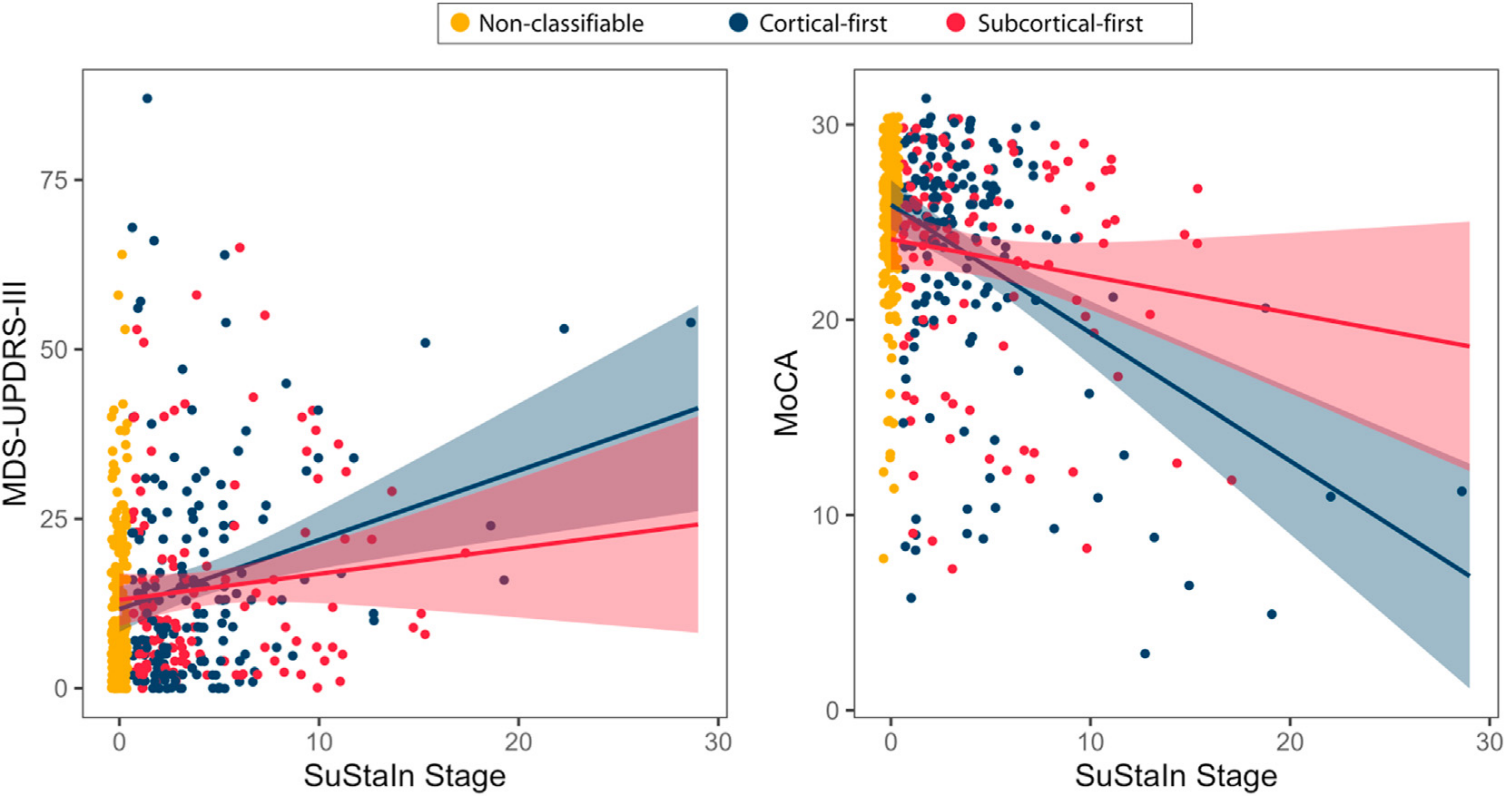
**Progression of clinical variables by SuStaIn stage**. Higher SuStaIn stages was associated with worse clinical scores on MDS-UPDRS-III and MoCA in patients. MoCA = Montreal Cognitive Assessment; MDS-UPDRS-III = Movement Disorders Society – Unified Parkinson's Disease Rating Scale, Part III; SuStaIn = Subtype and Staging Inference.

To account for disease progression inside subtypes, we next sought to determine whether global cognition and parkinsonian motor features varied as a function of SuStaIn subtype and severity. Using linear regression to predict MoCA scores based on age, sex, SuStaIn stage and subtype (and its interaction), and probability of subtype, we found significant effects of age and interaction between SuStaIn subtype and stage (Fig. 4 and Table 2). Higher age was associated with lower MoCA scores (estimate [95% CI] = −0.37 [−0.46, −0.27], p < 0.001 [linear regression]). The interaction effect indicated that SuStaIn stage had a steeper negative impact on MoCA scores in patients classified as cortical-first compared to those classified as subcortical-first. Specifically, in cortical-first patients, there was a significant negative correlation between SuStaIn stage and MoCA scores (r = −0.28, corrected for age, sex, and subtype probability, p < 0.001 [Pearson's correlation]), whereas no significant relationship was observed in subcortical-first patients (r = 0.002, corrected for age, sex, and subtype probability, p = 0.98 [Pearson's correlation]). In contrast, when predicting MDS-UPDRS-III scores, the analysis revealed significant effects of age and SuStaIn stage only. Older age (estimate [95% CI] = 0.92 [0.66, 1.17], p < 0.001 [linear regression]) and higher SuStaIn stage (atrophy progression) (estimate [95% CI] = 2.31 [0.09, 4.52], p = 0.041 [linear regression]) were both significantly associated with increased MDS-UPDRS-III scores (Fig. 4 and Table 2). Taken together, cognitive decline (as measured by MoCA) was more strongly associated with disease progression in cortical-first patients, while motor impairment (as measured by MDS-UPDRS-III) was influenced by both age and overall disease stage, regardless of subtype.

**Table 2:** Associations between clinical variables and SuStaIn subtype and stage.

Variable	Estimate	Standard error	t-value	p-value^a^	95% CI for estimate
***MoCA***					
Age	**−0.365**	**0.048**	**−7.350**	**<0.001**	**−0.460 to −0.269**
Sex^b^	−1.833	0.981	−1.868	0.063	−3.766 to 0.100
SuStaIn subtype^c^	−1.269	1.023	−1.240	0.216	−3.285 to 0.747
SuStaIn stage	**−1.178**	**0.419**	**−2.807**	**0.005**	**−2.004 to −0.351**
SuStaIn subtype ∗ stage	**0.383**	**0.170**	**2.253**	**0.025**	**0.048–0.717**
Probability of subtype assignment	**6.134**	**2.460**	**2.494**	**0.013**	**1.287–10.981**
Constant	**49.144**	**4.864**	**10.104**	**<0.001**	**39.561–58.728**
***MDS-UPDRS-III***					
Age	**0.916**	**0.130**	**7.066**	**<0.001**	**0.660–1.171**
Sex^b^	−0.020	2.562	−0.008	0.994	−5.069 to 5.028
SuStaIn subtype^c^	1.767	2.736	0.646	0.519	−3.624 to 7.158
SuStaIn stage	**2.307**	**1.125**	**2.050**	**0.041**	**0.090–4.524**
SuStaIn subtype ∗ stage	−0.806	0.455	−1.772	0.078	−1.702 to 0.090
Probability of subtype assignment	−12.815	6.501	−1.971	0.050	−25.624 to −0.006
Constant	**−44.782**	**12.743**	**−3.154**	**<0.001**	**−69.888 to −19.675**

Bold values represent significant p-values. MoCA = Montreal Cognitive Assessment; MDS-UPDRS-III = Movement Disorders Society – Unified Parkinson's Disease Rating Scale, Part III; SuStaIn = Subtype and Staging Inference.

a Separate regression models of clinical variable ∼ age + sex + subtype + stage + subtype ∗ stage + probability of subtype.

b Sex is coded as 0 = male and 1 = female.

c Subtype is coded as 0 = cortical-first subtype and 1 = subcortical-first subtype.

### Atrophy subtypes relate differently to phenoconversion in iRBD

We investigated whether SuStaIn atrophy subtypes were associated with phenoconversion risk and phenoconversion phenotypes in iRBD. Among all patients with iRBD, the mean follow-up time was 5.25 ± 3.34 years and 88 (24%) phenoconverted to a defined synucleinopathy, with 26 (30%) having developed DLB, 56 (63%) Parkinson's disease, and 6 (7%) multiple system atrophy. Although the unadjusted number of phenoconverted patients did not differ significantly between those classified within the SuStaIn subtypes (cortical-first or subcortical-first) and those who were not classified (stage 0/non-classifiable) (Supplementary Table S5), we performed a logistic regression to predict the log-odds of phenoconversion in iRBD based on age, sex, SuStaIn classifiability, SuStaIn stage, and the interaction between classifiability and stage (Supplementary Table S6). This analysis revealed a significant effect of classifiability on phenoconversion risk, with the log-odds of phenoconverting compared to remaining disease-free being significantly higher for patients with iRBD classified as subcortical-first compared to stage 0/non-classifiable patients (estimate [95% CI] = 2.56 [1.04–6.34], p = 0.042 [logistic regression]). Specifically, patients with iRBD within the subcortical-first subtype had 2.6 times higher odds of phenoconversion compared to remaining disease-free than those who were stage 0/non-classifiable.

**Fig. 5:**
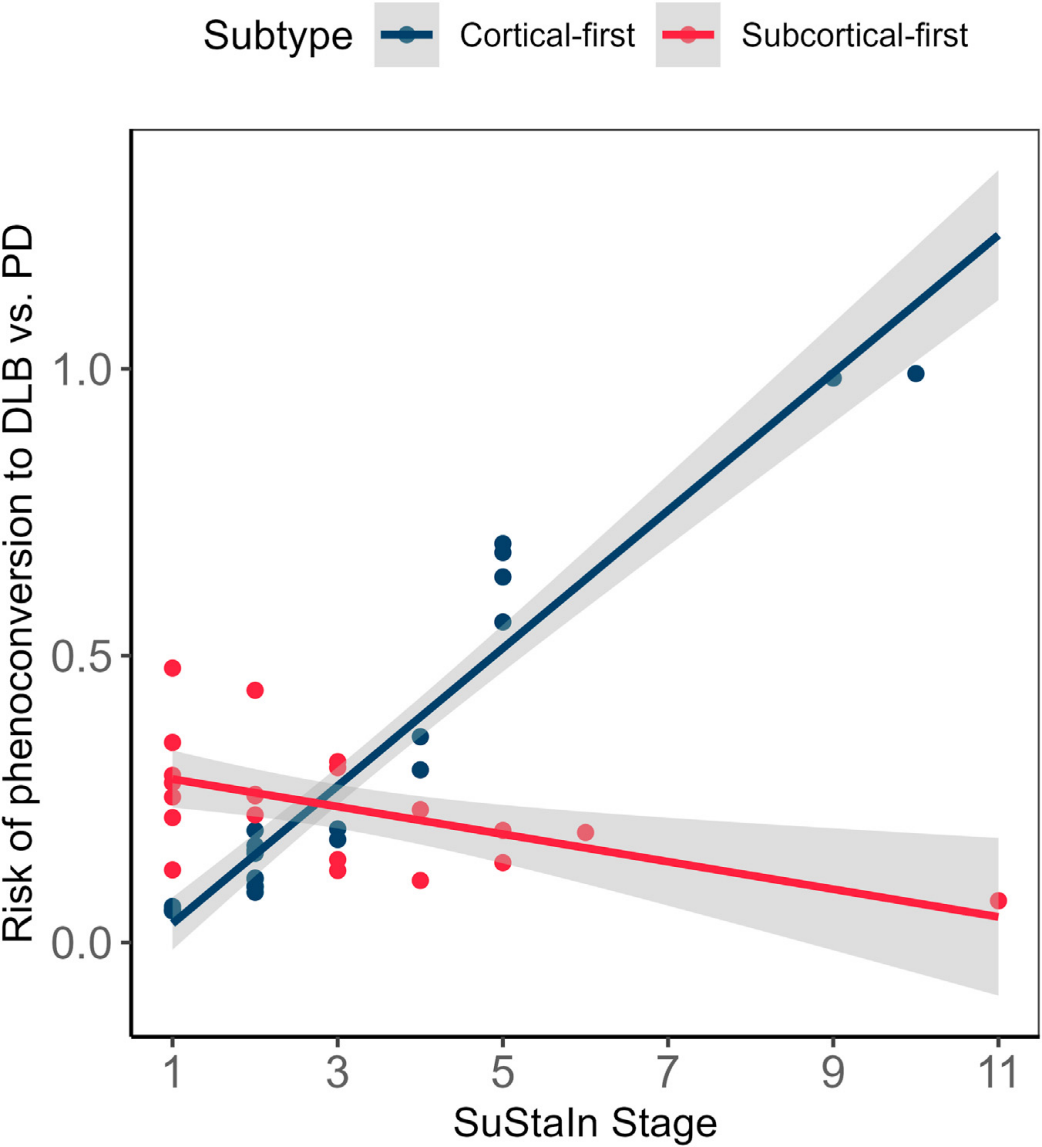
**Phenoconversion risk (calculated from the logistic regression predicting phenoconversion) differs in patients with iRBD based on classifiability and stage**. Patients with iRBD classified in the cortical-first subtype had a stronger likelihood of DLB compared to PD as disease severity (atrophy) increases. iRBD = idiopathic/isolated REM sleep behaviour disorder; SuStaIn = Subtype and Staging Inference.

We then investigated whether SuStaIn subtypes could predict the development of a parkinsonism or dementia-first phenotype in patients with iRBD while they were still in the preclinical stage, as predicting differential pathways in patients with iRBD who phenoconvert to DLB or PD is essential for developing a prognostic subtyping approach. Logistic regression to predict the log-odds of phenoconversion to DLB versus PD based on age, sex, SuStaIn subtype, SuStaIn stage, and their interaction revealed a significant interaction effect between SuStaIn subtype and SuStaIn stage (estimate [95% CI] = 0.36 [0.14, 0.93], p = 0.035 [logistic regression]) (Fig. 5, Supplementary Table S7). This interaction indicated that the log-odds of DLB compared to PD became more negative as both predictors increased. Specifically, unlike patients with iRBD classified within the subcortical-first subtype, the log-odds of DLB compared to PD in cortical-first patients increased as a function of SuStaIn stage (atrophy progression). Adding stage 0/non-classifiable patients with iRBD to the analysis further confirmed the association between higher SuStaIn stages and the likelihood of developing DLB rather than PD in patients with cortical-first iRBD (estimate [95% CI] = 0.35 [0.14, 0.90], p = 0.029 [logistic regression]). In other words, higher SuStaIn stages were associated with a greater likelihood of phenoconversion to DLB rather than PD in patients with iRBD classified as cortical-first.

## Discussion

In this study, we used a data-driven approach to identify two distinct patterns of brain atrophy progression, summarising the spectrum linking iRBD to overt disease. The first is a cortical-first atrophy progression subtype, where atrophy initially spreads throughout cortical areas before manifesting in subcortical structures later. The second is a subcortical-first atrophy progression subtype, where atrophy begins in the amygdala and basal ganglia before spreading to the cortical areas. Patients classified in the subtypes had an increased clinical burden compared to patients not subtyped by our modelling. Clinical scores of disease severity worsened with increasing stages of atrophy, with the progression of parkinsonian motor features increasing regardless of whether patients were classified as cortical- or subcortical-first subtypes. However, cognitive decline was specific to patients classified within the cortical-first phenotype. Phenoconversion trajectories also differed based on the subtype, with patients with iRBD with a cortical-first atrophy subtype being more likely to phenoconvert to DLB, while this pattern was not observed in patients classified as subcortical-first. Our results provide insights into the progression of brain atrophy in prodromal synucleinopathy as it develops towards manifest disease, which may have potential utility for prognostication and patient stratification.

SuStaIn is an unsupervised machine learning model developed to untangle the complexity of neurodegenerative diseases by identifying distinct subtypes and staging their progression over time. In simple terms, SuStaIn works by simultaneously clustering patients based on patterns of brain atrophy and ordering these changes into a sequence of stages, thereby separating phenotypic (subtype) differences from temporal (stage) progression. SuStaIn makes predictions by comparing an individual's biomarker values (in this instance, select regions of brain atrophy) to its learnt progression patterns—if a region's z-score crosses a specific threshold, it indicates a transition to a later stage and helps assign the patient to a particular subtype, thus providing a straightforward decision rule for stratifying patients based on disease severity and pattern. Once fully established, SuStaIn-based prediction could be applied at the individual level by deriving region-specific progression slopes and assessing the similarity of a given brain scan to each subtype. This would allow for classifying individual scans into the likeliest progression subtype, enabling better stratification of iRBD patients based on actual neurodegeneration and predicted disease trajectory. Additionally, it could serve as a benchmark to assess whether a patient's brain disease progression follows the expected trajectory for their subtype or deviates from it. However, before clinical implementation, further studies are needed to validate these subtypes in relation to other biomarkers, including clinical, biofluid, and genetic markers. This will help refine their predictive value and ensure their utility for patient monitoring and trial stratification.

Previous studies have found cortical and subcortical atrophy in patients with iRBD, which have been shown to correlate with motor and cognitive dysfunction, as well as predict phenoconversion to dementia.^5^^,^^7^, ^8^, ^9^^,^^15^^,^^38^ The atrophy in iRBD, as in several neurodegenerative diseases,^13^^,^^39^^,^^40^ has been shown to be constrained by both the brain's structural connectivity pattern and the local patterns of gene expression,^5^ targeting preferentially regions overexpressing genes involved in energy production and protein degradation.^6^ Distinct patterns of cortical and subcortical atrophy have also been described in patients with mild cognitive impairment who later developed DLB.^41^ Patients with DLB similarly show unique patterns of atrophy when compared with patients with Alzheimer's disease and healthy controls,^42^^,^^43^ with a hippocampal-sparing pattern of regional atrophy observed in DLB, which may be influenced by mixed co-pathology.^44^ The distinct involvement of brain structures at earlier and later disease stages depending on machine learning-derived subtypes has also been described in individuals with manifest Parkinson's disease.^45^ The broad areas and patterns of atrophy in prodromal synucleinopathy and overt disease found in the present study are in line with these results. Here, using a large cross-sectional sample size of brain MRI scans in iRBD, PD-pRBD, and DLB and machine learning, we were able to account for the variability in disease stage across individuals and reconstruct the progression of atrophy even at very early stages of disease. Our results not only support the finding that atrophy is diffuse in the late stages of synucleinopathy, but also suggest that the origin and pathway towards this state follows distinct patterns. These different patterns of atrophic spread, based solely on the data-driven analysis of quantitative atrophy derived from brain MRI scans, could have relevance for prognosis or more precisely select patients for disease-modifying trials. For this to be the case, future studies will need to derive signature patterns for each of these subtypes and develop algorithms that will allow classifying brain MRI scans from patients into the likeliest subtype.

The identified subtypes were significantly associated with clinical features and progression trajectories. Indeed, we observed that higher SuStaIn stages within subtypes, reflecting more advanced brain disease (atrophy) progression, were associated with worse clinical scores. Furthermore, both cortical-first and subcortical-first subtypes were associated with a higher rate of increase in MDS-UPDRS-III scores over time, aligning with parkinsonian motor features being common to patients with iRBD progressing to either DLB or PD. Indeed, both DLB and PD phenoconverters have similarly elevated MDS-UPDRS-III in the iRBD stage, and the motor interval is, if anything, longer in DLB phenoconverters than PD phenoconverters.^46^ However, in contrast, cognitive decline measured by MoCA was subtype-specific, being associated with advancing SuStaIn stages in patients classified as cortical-first but not in those classified as subcortical-first. This supports the idea that cortical-first patients show a closer association between cognitive and parkinsonian features, aligning with a trajectory toward DLB. Notably, atrophy in the posterior cortical region was affected at late SuStaIn stages in the cortical subtype, in keeping with the fact that visuospatial dysfunction is a harbinger of phenoconversion to DLB.^10^ Importantly, the MDS-UPDRS-III and MoCA are broad metrics of motor and cognitive function, which do not fully capture the breadth or depth of dysfunction in iRBD.^46^^,^^47^ Future work shall examine if different subtype progression patterns are associated with more specific patterns of clinical dysfunction.

**Fig. 6:**
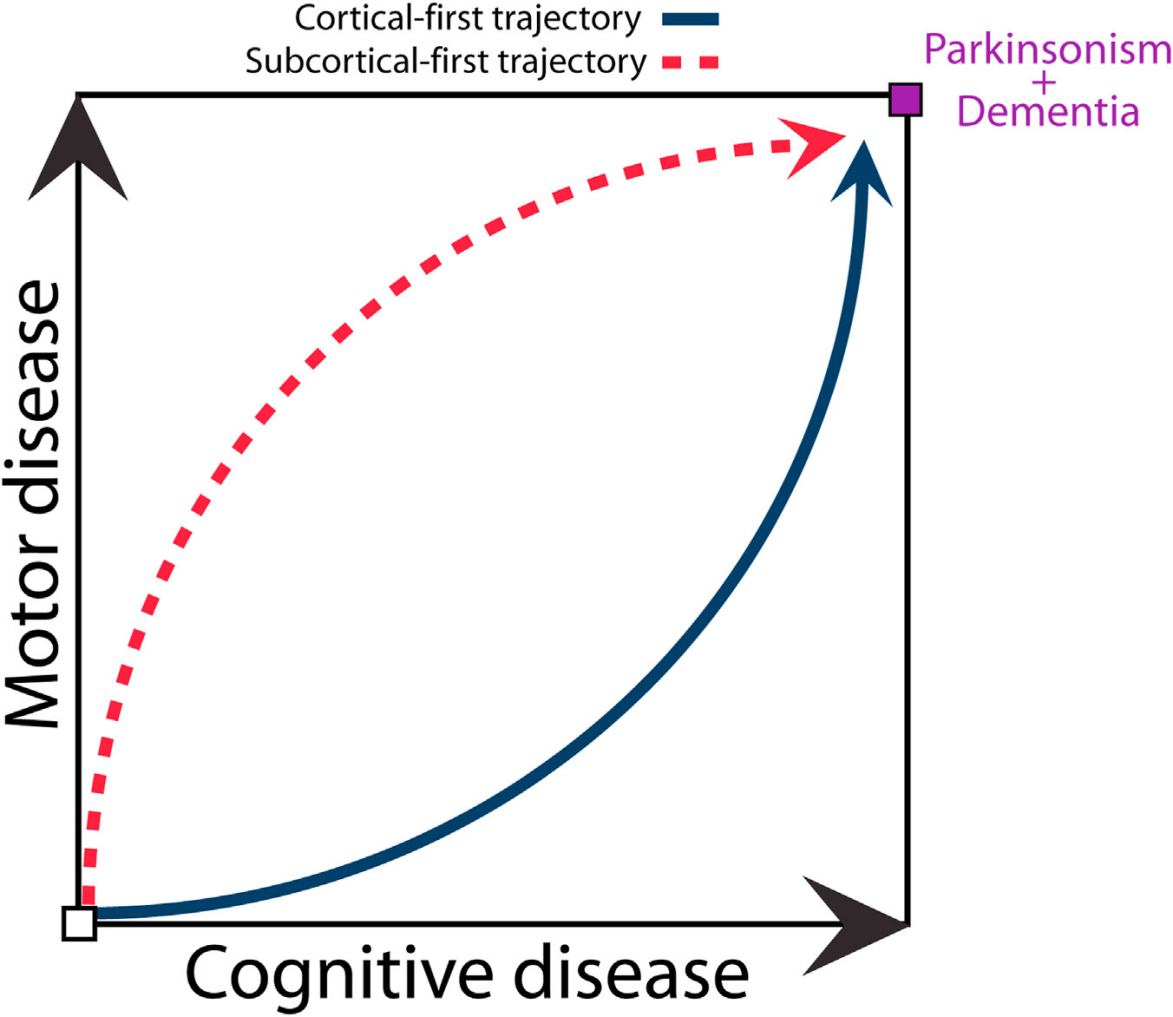
**Hypothetical schematic representing the pathways of evolution of brain atrophy progression in iRBD, as simulated by SuStaIn**. In this model, the subcortical-first subtype is associated with increased phenoconversion compared to non-classified patients, possibly due to initial involvement of the basal ganglia structures. By contrast, the cortical-first subtype is associated with specific phenoconversion to DLB compared to PD as disease severity (atrophy) progresses. This model suggests that the cortical-first subtype is more closely related to what is classically known as DLB (i.e., initial cortical involvement followed by subcortical involvement, with a long-term risk of dementia), whereas the subcortical-first subtype is more closely related to PD (i.e., initial subcortical involvement followed by cortical involvement, with earlier phenoconversion to PD and an increased long-term risk of dementia under the label of PD dementia). DLB = dementia with Lewy bodies; iRBD = idiopathic/isolated REM sleep behaviour disorder; PD = Parkinson's disease.

Regression analyses indicated that classifiable subjects with iRBD had a higher risk of phenoconversion than stage 0/non-classifiable subjects. The subcortical-first brain atrophy progression subtype in iRBD was associated with a stronger likelihood of developing an overt synucleinopathy. We propose that as subcortical structures are affected initially, the hallmark clinical features of parkinsonism become manifest, leading to a diagnosis earlier in the subcortical-first subtype. In contrast, the cortical-first phenotype remains “healthier” (disease-free) for longer periods until subcortical structures become involved, at which point motor signs and symptoms of disease appear, and phenoconversion occurs. In other words, whereas both subtypes are associated with parkinsonian motor features with increasing progression, the cortical-first phenotype is more strongly associated with cognitive decline and the development of DLB compared to PD over time in iRBD (Fig. 6). This may indicate that the cortical-first subtype is more closely related to what is classically known as DLB (i.e., initial cortical involvement followed by subcortical involvement, with a long-term risk of dementia), whereas the subcortical-first subtype is more closely related to PD (i.e., initial subcortical involvement followed by cortical involvement, with earlier phenoconversion to PD and an increased long-term risk of dementia under the label of PD dementia).

Several hypotheses may explain the pathophysiological patterns of each subtype. First, the patterns of atrophy may be reflective of ongoing neurodegeneration in different regions of the brain in iRBD: the cortical-first subtype begins with neurodegeneration of the cortex while the subcortical-first subtype begins with atrophy in the limbic and basal ganglia structures. It is important to keep in mind that our model was built on atrophy and not actual pathology, and that although preliminary evidence has shown that atrophy in synucleinopathies can be recreated *in silico* as a spread of alpha-synuclein misfolded proteins,^5^ several other proteins and co-pathologies may also be at play in iRBD-associated neurodegeneration. For example, brain neurodegeneration in DLB patients is associated with amyloid beta and tau deposition at baseline^48^^,^^49^ and lower CSF levels of amyloid beta 42 have been found in iRBD compared to controls.^50^^,^^51^ Moreover, 25% of patients with iRBD are found to be amyloid beta-positive.^52^ Future studies should investigate whether the cortical- and subcortical-first atrophy subtypes of iRBD differ on imaging and blood- and CSF-based markers of Alzheimer's disease co-pathologies. Another possibility is that the cortical-first subtype represents a relatively “resilient” subtype compared to the subcortical-first subtype, where pathology also spreads through the subcortical structures but do not manifest as observable and quantifiable atrophy, unlike cortical areas. From this angle, both subtypes would have the same initial starting point, and patients within the subcortical-first subtype would represent increased vulnerability of the basal ganglia structures in showing neurodegeneration and displaying atrophy. While SuStaIn stages reflect a temporal sequence of events (allowing us to use terms like “earlier” and “later” stages), it cannot necessarily inform us about the actual timing or speed of the events. As such, we do not suggest that the subcortical group necessarily had a “faster” time to phenoconversion—rather, the timing of atrophic changes in the subcortical structures (earlier in some patients with iRBD, later in others) is what appears to determine the development of the key features of parkinsonism *vs*. dementia. Importantly, patients with iRBD classified in the cortical- or subcortical-first subtypes did not significantly differ in age, suggesting that age-related effects on SuStaIn modelling are unlikely to explain the observed differences in progression patterns. Otherwise, despite patients with iRBD being classified in this study as cortical- or subcortical-first, previous models have demonstrated that the iRBD phenotype belongs to a body-first propagation of pathology compared to a brain-first (i.e., pathology spreading from the gut to the brain and not from the brain to the gut).^53^ Therefore, it could be that the impact of pathology differs between subtypes, with the cortical-first subtype impacting more strongly upon several brainstem nuclei and neurotransmitter systems whose upstream changes yield observable morphological changes. Finally, it may be that deviations in morphological measurements compared to what was expected for age and sex are reflective of long-term genetic, lifestyle and environmental factors, which render the brain differently vulnerable to synucleinopathies once the pathological process hits.

Approximately half of the patients with iRBD were not classifiable into a disease subtype. The stage 0/non-classifiable patients were significantly younger, had better MDS-UPDRS-III and MoCA scores, and less overall brain atrophy. This was expected, as previous studies using computational neuroimaging in iRBD demonstrated that cognitive impairments account for a large variance of the morphological changes associated with iRBD,^8^ being significantly more prominent in the presence of mild cognitive impairment,^7^ which affects 30%–50% of patients with iRBD.^10^^,^^54^ A smaller number of patients with DLB (23%) were stage 0/non-classifiable, similar in proportion to a recent SuStaIn study in patients with progressive supranuclear palsy.^29^ Patients with DLB that were stage 0/non-classifiable were also younger relative to classifiable patients with DLB and had better MoCA scores, although all met criteria for dementia. It is possible that stage 0/non-classifiable patients with iRBD or DLB reflect phenotypes with less overall disease burden or perhaps different patterns of brain atrophy progression. For example, it is known that patients with *de novo* DLB have much higher frequencies of Alzheimer's disease co-pathology as compared to patients with *de novo* Parkinson's disease, implying that Alzheimer's disease co-pathology during the prodromal phase is a strong determinant of DLB phenoconversion and the development of dementia in synucleinopathies in general.^55^ Information about Alzheimer's disease co-pathology was not available for this study; however, we speculate that the unclassifiable patients with DLB or iRBD could reflect a “pure” alpha-synuclein phenotype with limited co-pathology, with consequently a lesser degree of atrophy. In keeping with this possibility, the presence of PD-pRBD—who had less overall atrophy and presumably less Alzheimer's disease co-pathology—to the SuStaIn model did not substantively change the subtyping patterns compared to when including only patients with iRBD or DLB, nor did it result in a significant number of previously stage 0/non-classifiable patients becoming classifiable or create a novel alternative subtype. Moreover, 50% of the patients with PD-pRBD were not classifiable, in keeping with the fact that atrophy was the primary driver of subtyping and staging. Once a computational framework becomes available for obtaining a probability of subtyping from individual brain MRI scans in iRBD, future studies should investigate more thoroughly the clinical features and biological underpinnings of classified and unclassified patients.

Some limitations in this study should be discussed. First, the modelling based on the SuStaIn algorithm recreated spatiotemporal brain atrophy progression patterns from cross-sectional MRI scans. Although powerful for leveraging large datasets of brain disease scans, future initiatives should aim at investigating the differential pathways of brain disease progression from longitudinal MRI scans in patients with iRBD. Second, even though this multicentric study involved the largest MRI sample of patients of polysomnography-proven iRBD, the number of patients remains limited, which increases the uncertainty of staging. Moreover, large regions of interest were used to better balance the spatial and temporal dimensions, which may have hidden the presence of atrophy in smaller areas. This is even more important for the brainstem, where specific nuclei have been reported to be impacted by neurodegeneration and pathology in iRBD.^56^, ^57^, ^58^ Another limitation of the study is the combination of patients from different centres including distinct imaging and acquisition protocols. However, we performed our analyses on imaging data that were harmonised for the effect of imaging site using NeuroComBAT. All DLB and PD diagnoses were made clinically and not confirmed at post-mortem; thus, some degree of misdiagnosis cannot be excluded, for example, with Alzheimer's disease. Furthermore, patients with DLB were also not polysomnography-proven to have RBD, although RBD is highly prevalent in DLB (70–90% of patients) and is a core clinical feature in the diagnosis.^22^ As RBD is less common in PD (25–58%),^59^ we explicitly included patients with PD-pRBD. We did not additionally include patients with PD and dementia (PDD), since any patient with PD-RBD who develops dementia meets diagnostic criteria for DLB.^22^ In order to harmonise clinical data, MMSE scores were converted to estimated MoCA scores in a subset of participants, which may limit the interpretation of cognitive function since the MMSE is less sensitive to mild cognitive impairment.^37^ However, the majority of such conversions involved those with DLB, who meet criteria for dementia by definition. Finally, due to limitations on available MRI studies, we were not able to verify subtyping patterns using an independent replication sample set; however, to our knowledge, our primary analysis has used by far the largest sample size of prodromal synucleinopathy MRIs assembled to date. The strength of this study from a generalisability perspective is that it reflects the combined experience of 11 international study centres and used controls from each. What limits the generalisability is the fact that most study subjects were Caucasian, and all study centres were in relatively higher-income countries, which is a common issue in prospective studies of this nature.

In conclusion, we demonstrate data-driven evidence for the existence of two atrophy progression subtypes in iRBD. The cortical-first subtype was associated with a greater likelihood of DLB over time in iRBD, while both the cortical- and subcortical-first subtypes were associated with increasing parkinsonian motor features over time. The accurate identification and staging of patients with iRBD may have important implications for tracking disease progression.

## Contributors

All authors read and approved the final version of the manuscript. Stephen Joza was involved in conceptualisation, data curation, formal analysis, and drafting the original manuscript.

Aline Delva, Christina Tremblay, Andrew Vo, Marie Filiatrault, and Max Tweedale contributed to imaging data curation and review and editing of the manuscript.

Jean-François Gagnon, Ronald B. Postuma, Alain Dagher, Johannes Klein, Michele Hu, Petr Dusek, Stanislav Marecek, Zsoka Varga, John-Paul Taylor, John T. O'Brien, Michael Firbank, Alan Thomas, Paul C. Donaghy, Stephane Lehericy, Isabelle Arnulf, Marie Vidailhet, Jean-Christophe Corvol, the ICEBERG Study Group, Jean-François Gagnon, Ronald B. Postuma, Alain Dagher, Richard Camicioli, Howard Chertkow, Simon Lewis, Elie Matar, Kaylena A. Ehgoetz Martens, Lachlan Churchill, Michael Sommerauer, Sinah Röttgen, Per Borghammer, Karoline Knudsen, Allan K. Hansen, Dario Arnaldi, Beatrice Orso, Pietro Mattioli, Luca Roccatagliata, and Oury Monchi were involved in clinical investigation, funding acquisition, and review and editing of the manuscript.

Shady Rahayel was involved in conceptualisation, data curation, formal analysis, funding acquisition, investigation, project administration, and review and editing of the manuscript.

To ensure data integrity, Stephen Joza, Christina Tremblay, and Shady Rahayel directly accessed and verified the underlying data reported in the manuscript.

## Data sharing statement

The data used in this study were obtained from multiple collaborating centres, each of which retains ownership of their respective datasets. The principal investigator had authorised access to all data necessary for the analyses performed in this study. However, the accessibility and sharing of data are subject to the local policies and restriction criteria of each centre involved. As such, data availability is restricted, and requests for access should be directed to the respective institutions, pending their specific data access and sharing guidelines. Qualified researchers may obtain access to all de-identified imaging data part of the PPMI or CCNA studies from their respective platforms. Source code for the pySuStaIn algorithm is available at http://github.com/ucl-pond/.

## Declaration of interests

Outside the submitted work, Stephen Joza received support for attending meetings and/or travel from the American Academy of Neurology and Parkinson's Canada. Jean-François Gagnon received funding from the NIH/NIA. Ronald B. Postuma received grants from the CIHR, Michael J. Fox Foundation, NIH, Roche Diagnostics, and the Weston Foundation. He received consulting fees from Novartis, Eisai, Merck, Vaxxinity, BMS, Ventus, Korro, Vanqua, Roche, Regeneron, Helicon, Epic, and Clinilabs. He holds leadership roles with Parkinson Canada, the Michael J. Fox Foundation, MDS, *Movement Disorders* journal, and the RBD Study Group. Alain Dagher received travel support from the Michael J. Fox Foundation. Johannes C. Klein receives salary support from the NIHR Oxford Health Clinical Research Facility and the NIHR Oxford BRC, speaker honoraria from Merz, Ipsen, and AbbVie, and travel reimbursement from Merz and Ipsen. Michele Hu received consulting fees from Lundbeck, ESCAPE Bio, Evidera, Manus Neurodynamica, Biogen MA, CuraSen Therapeutics, Roche, JAZZ Pharma, and Aventis Pharma. She received honoraria and support for attending meetings from the International Movement Disorders Society, the 10th Singapore International Parkinson Disease and Movement Disorder Symposium, and the World Parkinson Congress. She holds a patent for predicting striatal dopamine levels via smartphone, serves on advisory boards and DSMBs including the Exenatide-PD3 Trial and ISAP Trial Steering Committee, is Treasurer of the ABN MDSIG, a shareholder and advisory founder of NeuHealth Digital Ltd. John T. O'Brien received consulting fees from Biogen and acted as a consultant for Roche, GE Healthcare, and Okwin. He received honoraria for lectures from GE Healthcare, serves on advisory boards or DSMBs for TauRx and Novo Nordisk, chairs the Research Strategy Council of the UK Alzheimer's Society, and received research support from Avid/Lilly, Merck, UCB, and Alliance Medical. Paul C. Donaghy received grants or contracts, paid to his institution, from Alzheimer's Research UK, the Michael J. Fox Foundation, the Alzheimer's Society, and GE Healthcare, and received an honorarium for a lecture at the Lewy Body Masterclass (paid to his institution). Jean-Christophe Corvol received grants/contracts from the Paris Brain Institute, ANR, and AXA Foundation (paid to institution), consulting fees from Roche, Servier, UCB, Ferrer, Alzprotect, iRegene, and Bayer, and serves on the Servier advisory board. Richard Camicioli serves (unpaid) on the Research and Scientific Advisory Board of Parkinson Canada. Howard Chertkow is the Scientific Director of CCNA (unpaid) and principal investigator or co-investigator on major research grants including from CIHR ($20.3M, 2024–29), Alzheimer's Society of Canada, BrightFocus, and NIH. He has also received funding for multi-site clinical trials sponsored by IntelGenX, Alector, Eli Lilly, Biogen, Hoffman LaRoche, and Anavex. He serves on advisory boards for Lilly and Eisai (personal payment). Simon Lewis received travel support from the International Parkinson's and Movement Disorder Society as a member of their Congress Scientific Program Committee (2022–2025). He holds leadership roles on editorial boards (*Translational Neurodegeneration*, *Journal of Parkinson's Disease*, *Movement Disorders*, *Parkinsonism and Related Disorders*, *Journal of Neurology*) and various international MDS working groups. Elie Matar received honoraria from CSL Seqirus and the International Parkinson's and Movement Disorders Society for presentations on non-motor and cognitive symptoms in Parkinson's. Dario Arnaldi received honoraria for lectures from Idorsia, Italfarmaco, PIAM, and Bruno. Beatrice Orso received a research grant from GE Healthcare. Shady Rahayel received grant support and travel reimbursement from the Michael J. Fox Foundation. Aline Delva, Christina Tremblay, Andrew Vo, Marie Filiatrault, Max Tweedale, John-Paul Taylor, Michael Firbank, Alan Thomas, Petr Dusek, Stanislav Marecek, Zsoka Varga, Stephane Lehericy, Isabelle Arnulf, Marie Vidailhet, Kaylena A. Ehgoetz Martens, Lachlan Churchill, Michael Sommerauer, Sinah Röttgen, Per Borghammer, Karoline Knudsen, Allan K. Hansen, Pietro Mattioli, Luca Roccatagliata, and Oury Monchi report no conflicts of interest.
