# Supplementary material for: Distinct brain atrophy progression subtypes underlie phenoconversion in isolated REM sleep behaviour disorder
Source: eBioMedicine. 2025 May 29;117:105753. doi: 10.1016/j.ebiom.2025.105753 (PMC12177146; doi:10.1016/j.ebiom.2025.105753)
Supplement: Supplementary Tables and Figures [file mmc1.docx]

**Supplementary Table 1 Sample size and MRI acquisition parameters by study centre**

| **Centre** | **Controls** | **iRBD** | **DLB** | **PD-pRBD** | **Scanner** | **Sequence** | **TR (ms)** | **TE (ms)** | **Flip angle** | **Voxel size** |
| --- | --- | --- | --- | --- | --- | --- | --- | --- | --- | --- |
| Aarhus | 19/20 | 13/18 | - | - | 3T Siemens  MAGNETOM Skyra  32c head coil | MPRAGE | 2420 | 3.7 | 9° | 1 mm³ isotropic |
| CCNA | 57/57 | - | 13/14 | - | See reference 18 | - | - | - | - | - |
| Cologne | - | 31/47 | - | - | 1.5T Philips  INGENIA  16c head coil | MPRAGE | 7.6 | 3.5 | 8° | 0.55 x 0.55 x 0.95 mm |
| Genoa | 13/15 | 10/14 | - | - | 3T Siemens  PRISMA  64c head coil | MPRAGE | 2300 | 2.98 | 9° | 1 mm³ isotropic |
| Montreal | 84/94 | 73/84 | - | - | 3T Siemens  TIM Trio  12c head coil  3T Siemens  MAGNETOM  Prisma  32c head coil | MPRAGE  MPRAGE | 2300  2300 | 2.91  2.98 | 9°  9° | 1 mm³ isotropic  1 mm³ isotropic |
| Newcastle | 59/62 | - | 69/73 | - | 3T Philips  Achieva  8c head coil  3T Philips  Achieva,  8c head coil | MPRAGE  MPRAGE | 8.3  9.6 | 4.6  4.6 | 8°  8° | 1 mm³ isotropic  1.15 x 1.15 x 1.2 mm |
| Oxford | 59/66 | 73/81 | - | - | 3T Siemens  Trio  12c head coil | MPRAGE | 2040 | 4.7 | 8° | 1 mm³ isotropic |
| PPMI | 92/126 | 17/37 | - | 110/142 | See reference 17 | - | - | - | - | - |
| Paris | 60/73 | 49/57 | - | - | 3T Siemens  TIM Trio  12c head coil  3T PRISMA  Fit  64c head coil | MPRAGE  MPRAGE2 | 2300  5000 | 4.18  2.98 | 9°  4° & 5° | 1 mm³ isotropic  1 mm³ isotropic |
| Prague | 55/57 | 75/83 | - | - | 3T Siemens  SKYRA  32c head coil | MPRAGE | 2200 | 2.4 | 8° | 1 mm³ isotropic |
| Sydney | 21/26 | 21/30 | - | - | 3T GE Discovery  MR750  8c head coil | BRAVO | 5800 | 2.6 | 12° | 1 mm³ isotropic |
| Total | 519/596 | 362/451 | 82/87 | 110/142 | 3T Siemens  MAGNETOM Skyra  32c head coil | MPRAGE | 2420 | 3.7 | 9° | 1 mm³ isotropic |

Sample sizes reflect the number of patients used in the SuStaIn modelling. The first number reflects the number of patients who passed quality control, whereas the second number reflects the total number of patients imaged.

CCNA = Canadian Consortium on Neurodegeneration in Aging; DLB = dementia with Lewy bodies; iRBD = idiopathic/isolated REM sleep behaviour disorder; PD-pRBD = Parkinson’s disease with possible REM sleep behaviour disorder; PPMI = Parkinson’s Progression Markers Initiative.

**Supplementary Table 2 Descriptive statistics of patients by group**

| **Variable** | **Controls** | **iRBD** | **DLB** | **PD-pRBD** | **p-value** | **Post-hoc testing** |
| --- | --- | --- | --- | --- | --- | --- |
| Age | 65.6 ± 10.1 | 67.1 ± 6.95 | 76.8 ± 6.45 | 62.1± 8.93 | **<0.001** | DLB > Controls = iRBD > PD |
| %male/% female | 59.6/41.4 | 86.7/13.3 | 70.7/29.3 | 71.3/28.7 | **<0.001** | Control = DLB = PD > iRBD |
| MoCA | 26.8 ± 2.36 | 25.7 ± 3.02 | 14.4 ± 5.46 | 25.9 ± 3.31 | **<0.001** | Controls > PD = iRBD > DLB |
| MDS-UPDRS-III | 2.28 ± 4.44 | 6.04 ± 5.57 | 32.1 ± 18.1 | 21.4 ± 9.61 | **<0.001** | DLB > PD > iRBD > Controls |

Groups were compared using ANOVA with Tukey HSD post-hoc tests for continuous variables.

Chi-squared tests were used with post-hoc pairwise testing for categorical variables.

DLB = dementia with Lewy bodies; iRBD = idiopathic/isolated REM sleep behaviour disorder; MoCA = Montreal Cognitive Assessment; MDS-UPDRS-III = Movement Disorders Society – Unified Parkinson’s Disease Rating Scale, Part III; PD-pRBD = Parkinson’s disease with possible REM sleep behaviour disorder; SD = standard deviation.

**Supplementary Table 3 MRI regions of interest used as inputs in the model**

| **MRI ROI type** | **Brain Areas** |
| --- | --- |
| **Cortical volume** | Thalamus |
|  | Caudate |
|  | Putamen |
|  | Pallidum |
|  | Hippocampus |
|  | Amygdala |
|  | Nucleus accumbens |
|  | Brainstem |
| **Cortical thickness** | Frontal lobe |
|  | Parietal lobe |
|  | Temporal lobe |
|  | Occipital lobe |
|  | Cingulate |
|  | Insula |

MRI regions of interest used in the analysis using the Desikan-Killiany atlas. To reduce the number of input features when modelling subtypes and preserve sufficient power, the labels of each individual parcellation were fused together inside FreeSurfer to yield lobar measurements of cortical thickness for the frontal, parietal, temporal, occipital, and cingulate lobes.

**Supplementary Table 4 Demographic and clinical variables in the iRBD + DLB only model (excluding PD-pRBD)**

|  | **Classifiable** | | | **Subtyped** | | |
| --- | --- | --- | --- | --- | --- | --- |
|  | **Non-classifiable** | **Classifiable** | **p-value^a^** | **Cortical-first** | **Subcortical-first** | **p-value^b^** |
| **Demographics** |  |  |  |  |  |  |
| n (%): iRBD | 183 (92.9) | 179 (72.5) | 0.833 | 112 (74.7) | 67 (69.1) | **0.001** |
| n (%): DLB | 14 (7.1) | 68 (27.5) | **<0.001** | 38 (25.3) | 30 (30.9) | 0.332 |
| Age: All | 66.3 (7.2) | 71.0 (7.7) | **<0.001** | 70.5 (7.7) | 71.7 (7.5) | 0.249 |
| Age: iRBD | 65.8 (7.1) | 68.5 (6.5) | **<0.001** | 68 (6.3) | 69.2 (6.8) | 0.235 |
| Age: DLB | 72.7 (5.7) | 77.6 (6.3) | **<0.001** | 78 (6.7) | 77.2 (5.8) | 0.577 |
| % male/% female | 83.8/16.2 | 83.8/16.2 | 0.960 | 82.7/17.3 | 85.6/14.4 | 0.611 |
| Stage^c^ (SD): All | 0 (0) | 4.8 (4.7) | **<0.001** | 4.5 (4.8) | 5.3 (4.6) | 0.209 |
| Stage^c^ (SD): iRBD | 0 (0) | 3.6 (2.9) | **<0.001** | 3.2 (2.1) | 4.3 (3.9) | **0.043** |
| Stage^c^ (SD): DLB | 0 (0) | 7.9 (6.7) | **<0.001** | 8.2 (7.7) | 7.5 (5.4) | 0.630 |
| **Clinical variables** |  |  |  |  |  |  |
| MDS-UPDRS-III (SD): All | 6.5 (7.8) | 14.6 (16.4) | **<0.001** | 15.6 (18.2) | 13.2 (13.1) | 0.236 |
| MDS-UPDRS-III (SD): iRBD | 5.1 (4.7) | 7.1 (6.3) | **<0.001** | 7.1 (6.5) | 7.0 (5.9) | 0.932 |
| MDS-UPDRS-III (SD): DLB | 25.2 (14.2) | 33.5 (18.6) | 0.073 | 38.7 (19.9) | 26.9 (14.5) | **0.006** |
| MoCA (SD): All | 25.6 (3.5) | 22.5 (6.3) | **<0.001** | 22.5 (6.7) | 22.5 (5.8) | 0.955 |
| MoCA (SD): iRBD | 26.1 (2.8) | 25.4 (3.2) | **0.040** | 25.4 (3.3) | 25.5 (2.9) | 0.855 |
| MoCA (SD): DLB | 17.7 (5.1) | 13.8 (5.4) | **0.048** | 12.9 (5.9) | 15.0 (4.4) | 0.112 |
| % MCI: iRBD^d^ | 35.6 | 46.3 | **0.041** | 50.0% | 48.5% | 0.649 |

Statistical differences were calculated using unpaired t-tests for continuous variables and chi-squared test for categorical variables.

^a^Non-classifiable group versus classifiable group.

^b^Cortical-first subtype versus subcortical-first subtype.

^c^Stage refers to SuStaIn stage.

^d^MCI as defined by ≤ 25/30 on MoCA; all DLB patients met criteria for dementia.

DLB = dementia with Lewy bodies; iRBD = idiopathic/isolated REM sleep behaviour disorder; MoCA = Montreal Cognitive Assessment; MCI = mild cognitive impairment; MDS-UPDRS-III = Movement Disorders Society – Unified Parkinson’s Disease Rating Scale, Part III; MoCA = Montreal Cognitive Assessment; PD-pRBD = Parkinson’s disease with possible REM sleep behaviour disorder; SD = standard deviation; SuStaIn = Subtype and Staging Inference.

**Supplementary Table 5 Phenoconversion outcomes in iRBD patients by SuStaIn subtypes**

|  | **Classifiable** | | | **Subtyped** | | |
| --- | --- | --- | --- | --- | --- | --- |
| **Phenoconversion** | **Non-classifiable** | **Classifiable** | **p-value^a^** | **Cortical-first** | **Subcortical-first** | **p-value^b^** |
| All converters | 42 | 46 | 0.763 | 24 | 22 | 0.835 |
| DLB converters | 14 | 12 | - | 7 | 5 | - |
| PD converters | 24 | 32 | - | 16 | 16 | - |
| MSA converters | 4 | 2 | - | 1 | 1 | - |

Statistical differences were calculated using chi-squared tests.

^a^Non-classifiable group versus classifiable group.

^b^Cortical-first subtype versus subcortical-first subtype.

DLB = dementia with Lewy bodies; iRBD = idiopathic/isolated REM sleep behaviour disorder; MSA = multiple system atrophy; PD = Parkinson’s disease; SuStaIn = Subtype and Staging Inference.

**Supplementary Table 6 Risk of phenoconversion in iRBD based on SuStaIn classifiability**

| **Variable** | **Estimate** | **Standard error** | **p-value** | **OR** | **95% CI for OR** |
| --- | --- | --- | --- | --- | --- |
| Age | 0.018 | 0.018 | 0.989 | 1.018 | 0.982 – 1.055 |
| Sex^a^ | 0.009 | 0.385 | 0.545 | 1.009 | 0.474 – 2.149 |
| SuStaIn classifiability^b^ | - | - | 0.108 | - | - |
| SuStaIn classifiability, cortex-first | -0.126 | 0.484 | 0.795 | 0.882 | 0.341 – 2.277 |
| **SuStaIn classifiability, subcortex-first** | **0.941** | **0.462** | **0.042** | **2.562** | 1.036 – 6.336 |
| SuStaIn stage | 0.331 | 0.411 | 0.420 | 1.393 | 0.622 – 3.116 |
| SuStain classifiability * stage | -0.163 | 0.156 | 0.298 | 0.850 | 0.625 – 1.155 |
| Constant | -2.181 | 1.237 | 0.078 | 0.113 | - |

Logistic regression model of phenoconversion ∼ age + sex + classifiability + stage + classifiability * stage

^a^Sex is coded as 1 = male and 2 = female

^b^Classifiability is coded as 0 = non-classifiable, 1 = classifiable as cortical-first, and 2 = classifiable as subcortical-first

CI = confidence interval; iRBD = idiopathic/isolated REM sleep behaviour disorder; OR = odds ratio; SuStaIn = Subtype and Staging Inference.

**Supplementary Table 7 Risk of specific phenoconversion in iRBD by SuStaIn subtypes**

| **Variable** | **Estimate** | **Standard error** | **p-value** | **OR** | **95% CI for OR** |
| --- | --- | --- | --- | --- | --- |
| Age | 0.022 | 0.067 | 0.743 | 1.022 | 0.896 – 1.167 |
| Sex^a^ | 0.665 | 1.293 | 0.607 | 1.945 | 0.154 – 24.518 |
| SuStaIn subtype^b^ | 3.081 | 1.708 | 0.071 | 21.787 | 0.766 – 619.333 |
| **SuStaIn stage** | **2.897** | **1.319** | **0.028** | **18.115** | **1.365 – 240.412** |
| **SuStaIn subtype * stage** | **-1.030** | **0.489** | **0.035** | **0.357** | **0.137 – 0.932** |
| Constant | -5.287 | 4.821 | 0.273 | 0.005 | - |

Logistic regression model of specific phenoconversion (parkinsonism or dementia) ∼ age + sex + subtype + stage + subtype * stage.

^a^Sex is coded as 0 = male and 1 = female.
^b^Subtype is coded as 0 = cortical-first subtype and 1 = subcortical-first subtype.

iRBD = idiopathic/isolated REM sleep behaviour disorder; SuStaIn = Subtype and Staging Inference.

**Supplementary Figure 1: Age distribution of participants included in the analysis**


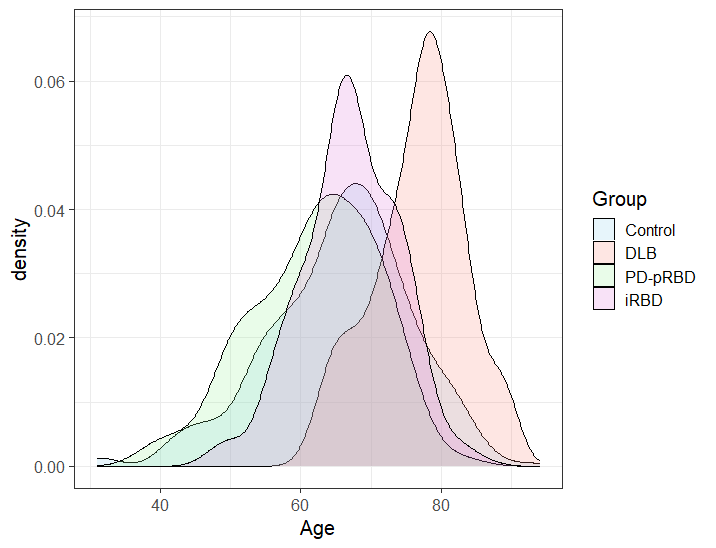


DLB = dementia with Lewy bodies; iRBD = idiopathic/isolated REM sleep behaviour disorder; PD-pRBD = Parkinson’s disease with probable REM sleep behavior disorder.

**Supplementary Figure 2: SuStaIn modelling using cortical volume and subcortical volume**


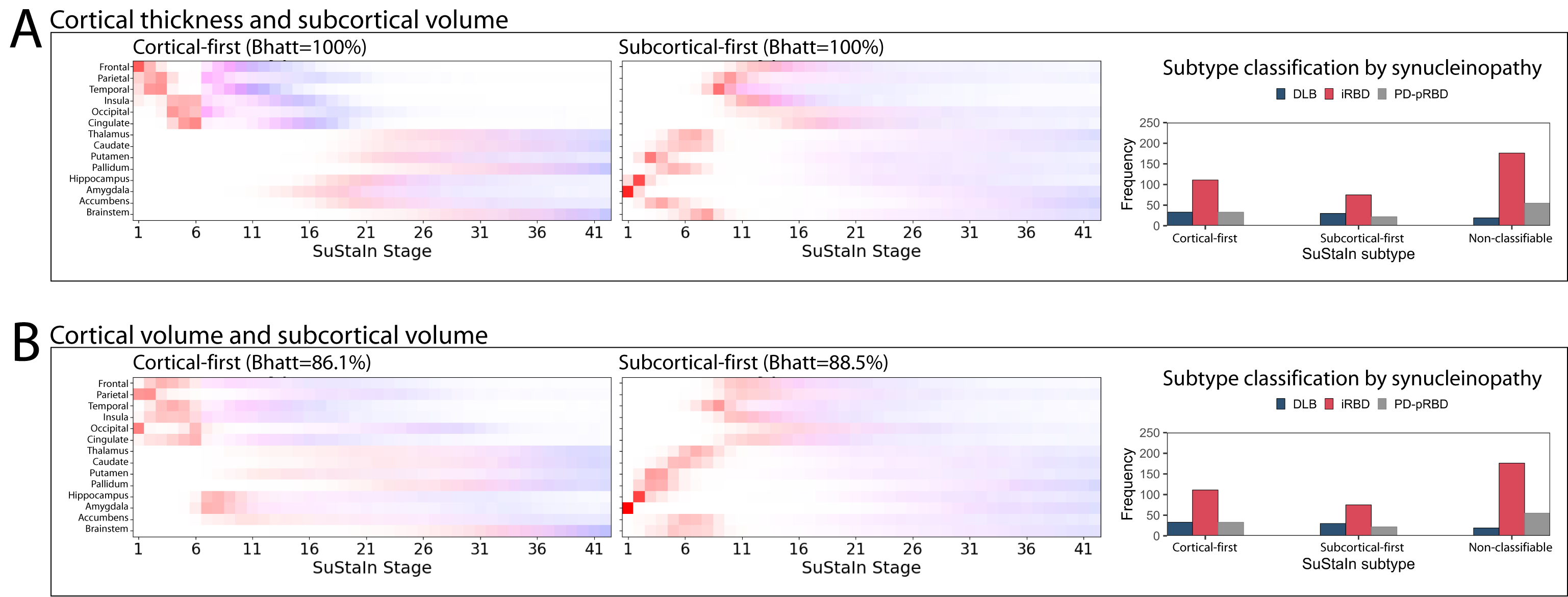


Positional variance diagram demonstrating two SuStaIn subtypes using **(A)** cortical thickness + subcortical volumes or **(B)** cortical volume + subcortical volumes as inputs into the model. In both models, SuStaIn identified two unique subtypes of brain atrophy progression with similar patterns of progression, as expressed by the Bhattacharyya coefficient. At each stage, the colour in each region indicates the level of severity of atrophy, with white representing unaffected regions, red mildly affected regions (z-score of -1), magenta moderately affected regions (z-score of -2), and blue severely affected regions (z-score of -3 or more). The number of patients that were classified into a subtype or remained unclassified are shown in the graphs on the right.

Bhatt = Bhattacharyya coefficient; DLB = dementia with Lewy bodies; iRBD = idiopathic/isolated REM sleep behaviour disorder; PD-pRBD = Parkinson’s disease with probable REM sleep behavior disorder; SuStaIn = Subtype and Staging Inference.

**Supplementary Figure 3: Effect of adding different synucleinopathies on the SuStaIn solution**


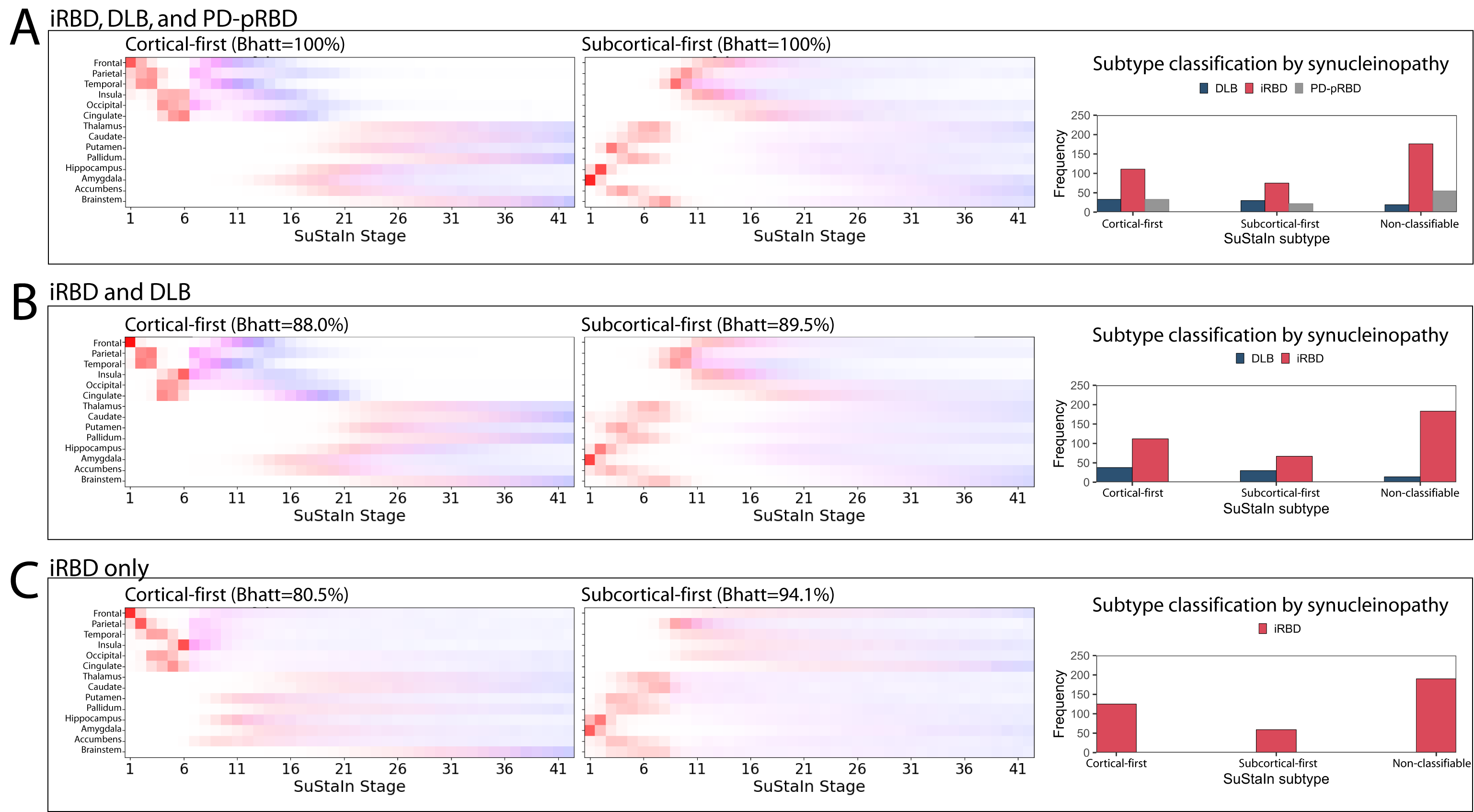


Positional variance diagrams comparing SuStaIn modelling using as input patients the **(A)** iRBD, DLB, and PD-pRBD patients – the primary model, **(B)** iRBD and DLB patients only, and **(C)** iRBD patients only. In each case, SuStaIn identified two unique subtypes of brain atrophy progression with similar patterns of progression as determined by the Bhattacharyya coefficient when comparing models (B) and (C) to the primary SuStaIn model used (A). At each stage, the colour in each region indicates the level of severity of atrophy, where white represents unaffected regions, red mildly affected regions (z-score of -1), magenta moderately affected regions (z-score of -2), and blue severely affected regions (z-score of -3 or less). Increased uncertainty of staging is observed at higher SuStaIn stages when DLB patients are not included in the modelling (“smudging”), resulting in a lower Bhattacharyya coefficient. The number of patients that were classified into a subtype or remained unclassified are shown in the graphs on the right.

Bhatt = Bhattacharyya coefficient; DLB = dementia with Lewy bodies; iRBD = idiopathic/isolated REM sleep behaviour disorder; PD-pRBD = Parkinson’s disease with probable REM sleep behaviour disorder; SuStaIn = Subtype and Staging Inference.
